# Non-toxic Polymeric Dots with the Strong Protein-Driven Enhancement of One- and Two-Photon Excited Emission for Sensitive and Non-destructive Albumin Sensing

**DOI:** 10.1021/acsami.2c08858

**Published:** 2022-08-26

**Authors:** Sebastian G. Mucha, Marta Piksa, Lucyna Firlej, Agnieszka Krystyniak, Mirosława O. Różycka, Wioletta Kazana, Krzysztof J. Pawlik, Marek Samoć, Katarzyna Matczyszyn

**Affiliations:** † Laboratoire Charles Coulomb, UMR5221, Université de Montpellier (CNRS), Montpellier 34095, France; ‡ Ludwik Hirszfeld Institute of Immunology and Experimental Therapy, Polish Academy of Sciences, Wroclaw 53-114, Poland; § Institute of Advanced Materials, Faculty of Chemistry, Wroclaw University of Science and Technology, Wroclaw 50-370, Poland; ∥ Department of Biochemistry, Molecular Biology and Biotechnology, Faculty of Chemistry, Wroclaw University of Science and Technology, Wroclaw 50-370, Poland

**Keywords:** polymer dots, non-toxic nanomaterials, serum albumins, two-photon excited fluorescence, fluorescence enhancement, albumin’s probe, nanomarkers, biosensing

## Abstract

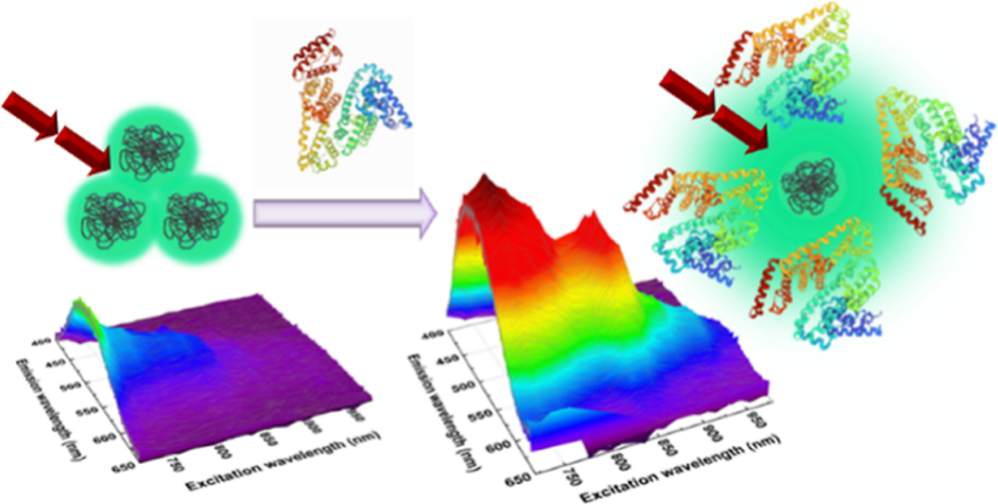

The need for efficient probing, sensing, and control of the bioactivity of biomolecules (e.g., albumins) has led to the engineering of new fluorescent albumins’ markers fulfilling very specific chemical, physical, and biological requirements. Here, we explore acetone-derived polymer dots (PDs) as promising candidates for albumin probes, with special attention paid to their cytocompatibility, two-photon absorption properties, and strong ability to non-destructively interact with serum albumins. The PDs show no cytotoxicity and exhibit high photostability. Their pronounced green fluorescence is observed upon both one-photon excitation (OPE) and two-photon excitation (TPE). Our studies show that both OPE and TPE emission responses of PDs are proteinaceous environment-sensitive. The proteins appear to constitute a matrix for the dispersion of fluorescent PDs, limiting both their aggregation and interactions with the aqueous environment. It results in a large enhancement of PD fluorescence. Meanwhile, the PDs do not interfere with the secondary protein structures of albumins, nor do they induce their aggregation, enabling the PD candidates to be good nanomarkers for non-destructive probing and sensing of albumins.

## Introduction

Serum albumins belong to a multigene family of extracellular plasma proteins in the vertebrates’ cardiovascular system, having numerous physiological functions. As the carrier proteins with multiple binding centers, serum albumins play a crucial role in the versatile transport and distribution of a variety of endogenous and exogenous chemical species, such as small organic compounds, long-chain fatty acids, metal ions, and others.^1−3^ Moreover, serum albumins account for the displacement of water molecules in the bloodstream, which influences the colloid osmotic pressure.^1^ The anomalies in the contents of albumins in the blood plasma (e.g., hypo- or hyperalbuminemia) can cause significant health issues, for instance, diabetes and cirrhosis, whose diagnosis is essential for the implementation of effective treatment.^4−6^ The most representative blood plasma proteins, human serum albumin (HSA) and bovine serum albumin (BSA), have been therefore extensively studied in terms of biochemical and pharmaceutical assays, including bioimaging,^7−9^ biosensing,^8,10^ and drug delivery systems.^11^

Sensing, probing, and control of the bioactivity of supramolecular agents upon external stimuli are currently being extensively developed. A particular importance is given to optical detection through such effects as absorption and one- and two-photon excitation (OPE and TPE, respectively) emission processes. In the case of HSA/BSA albumins, considerable attention has been paid to the study of interactions between proteins and optically active agents (probes) such as organic molecules,^12−15^ supramolecular assemblies,^16^ inorganic nanostructures,^8,17−23^ metallic nanoparticles,^24,25^ and carbon dots.^26,27^

The essential biological, chemical, and optical aspects that have to be considered when designing relevant optical, fluorescent probes are listed below:^16,28^

(i) They should provide remarkable OPE and TPE fluorescence activity.
(ii) They should absorb/emit in the red to near-infrared (NIR) wavelength range (the first optical window).^29^
(iii) The probe’s fluorescence response toward the titrating albumins’ sample should be strong and clear to enable a facile detection.
(iv) They need to form highly stable dispersions/solutions in aqueous media at physiological conditions and exhibit resistance toward ionic species.
(v) For medical and pharmaceutical applications, they should be cytocompatible and photostable.
(vi) Ideally, novel luminescent agents should be fabricated by simple, efficient, and cost-effective methods.

In the present paper, we focus on the merits of the use of a specific class of carbon-based nanomaterials, polymer dots (PDs), as fluorescent probes. Various carbon dots have been extensively explored over the last 2 decades and have become promising alternatives for organic dyes and inorganic semiconductor nanomaterials due to their interesting optical properties, long-term colloidal stability, excellent biocompatibility, low cytotoxicity, high photostability, and low-cost fabrication routes. Among the vast family of carbon dots, luminescent PDs are specific due to their mostly amorphous internal design. In contrast to graphitic carbon nanomaterials, PDs are usually composed of aliphatic chains, rich in diverse polar moieties, and assembled in a spherical shape by effective cross-linking, involving covalent bonding as well as weak interactions (e.g., hydrogen bonding and van der Waals forces). Recently, we reported a new, gram-scale synthesis route to produce acetone-derived hydrophilic and hydrophobic PDs featuring bright greenish-blue excitation-dependent emission and long fluorescence lifetimes (on the order of nanoseconds).^30^ In this work, we report on the cytocompatibility, the photostability, and the two-photon absorption (TPA) properties of the hydrophilic fraction of these PDs, which we consider to be promising luminescent agents. We investigated the interactions between PDs and both serum albumins in colloidal solutions using both OPE and TPE fluorescence techniques. In the different physiological conditions, substantial enhancements of the fluorescence of PDs in the presence of globular proteins were observed.

## Experimental Section

### Materials

Urea, deuterium oxide, sodium chloride, potassium chloride, sodium phosphate dibasic, monobasic potassium phosphate, sodium cacodylate, iron (II) chloride, copper (II) chloride, magnesium chloride, calcium chloride, sodium hydroxide, and hydrochloric acid were purchased from Sigma-Aldrich. Lysozyme (LYS), ovalbumin (OVA), HSA, and BSA in lyophilized powder forms were also purchased from Sigma-Aldrich. Acetone-based PDs were fabricated following the mechanism of the base-induced aldol reaction. The synthesis route and studies of their linear optical and structural properties were reported in a previous paper.^30^ Stock solutions of **C1K** and **C1Na** (for samples’ notation, see the Supporting Information) were prepared in Milli-Q water to reach the concentration of 0.5 mg/mL. The pH values of these samples were 7.2 and 7.3, respectively. To obtain the concentration of 10 mM and pH values equal to 7.2 (sodium cacodylate) and 7.4 (phosphate-buffered saline, PBS), two buffer solutions in Milli-Q water were prepared. BSA and HSA were dissolved in three different aqueous solutions: in Milli-Q water (pH = 7.0), in PBS, and in cacodylate buffer, to obtain final concentrations of 1 mM. The concentration values and the purity of proteins were examined using UV–vis absorption spectroscopy. High-glucose Dulbecco’s modified Eagle’s medium (DMEM) and trypsin–ethylenediaminetetraacetic acid were provided by the Laboratory of General Chemistry of the Institute of Immunology and Experimental Therapy, PAS (Wroclaw, Poland). Fetal bovine serum (FBS), l-glutamine, and antibiotics (penicillin/streptomycin mixture) were purchased from BioWest (Nuaillé, France). Bacterial lipopolysaccharide (LPS) from *E. coli* (serotype 055:B5) and 3-(4,5-dimethylthiazol-2-yl)-2-5-diphenyltetrazolium bromide (MTT) were obtained from Sigma (St. Louis, MO, USA).

### Steady-State Spectroscopy Characterizations

The UV–vis extinction spectra were recorded on a JASCO V-730 spectrophotometer in the 200–800 nm wavelength range, equipped with a Peltier thermo cell holder to adjust and control the temperature (5–70 °C). To provide the relevant temperature, the sample was kept in the cell holder for 300 s before each scan. Changes in OPE emission spectra were measured on a FluoroMax-4 spectrofluorometer (Horiba Jobin Yvon) for the selected excitation wavelengths of 350, 377, and 450 nm.^30^ The OPE excitation–emission maps of free PDs were determined using the FluoroMax-4 spectrofluorometer. To estimate absolute fluorescence quantum yields (FQYs), emission and excitation signals were collected on a custom-built setup, consisting of an integrating sphere, an FLS 980 Edinburgh Instruments spectrometer, and a BDL-375-SMN picosecond laser diode (377 nm). The abovementioned fluorescence spectroscopic measurements were performed at room temperature. The temperature-dependent OPE emission spectra were recorded upon the excitation at 350, 377, and 450 nm on an FS5 spectrofluorometer (Edinburgh Instruments). The experimental setup was equipped with a temperature-controlled sample holder (SC-25) and a TC 1 temperature controller (Quantum NorthWest), enabling the temperature to be tuned from 5 to 70 °C.

### Time-Resolved Spectroscopy Characterizations

Changes in fluorescence decays of PDs upon protein addition were recorded using a time-correlated single-photon counting (TCSPC) setup (Becker&Hickl GmbH) that contains an Acton SpectraPro SP-2300 monochromator (Princeton Instruments) and a high-speed hybrid detector HPM-100-50 (Becker&Hickl GmbH) combined with a DCC-100 card. A BDL-375-SMN picosecond laser diode (20 MHz, 377 nm) was used as the excitation source. Each fluorescence decay curve was averaged over six accumulations at room temperature.

### Multi-Photon Spectroscopy Experiments

Two-photon excited emission spectra were acquired on a home-built experimental setup, consisting of a Shamrock 303i spectrometer (Andor) equipped with a sensitive iDus camera (Andor) and a femtosecond laser system (Ti/sapphire Chameleon laser, Coherent Inc.) with the repetition rate of 80 MHz and the pulse duration of ∼120 fs, operating in the range 700–1000 nm. The laser beam power was monitored using a PM100D handheld optical power and energy meter (Thorlabs). To minimize undesired effects due to re-absorption and inner filter effects, the fluorescence was measured for samples at concentrations corresponding to absorbance values below 0.1 in the excitation and emission regions. Resistance to photobleaching was evaluated by monitoring OPE fluorescence spectra at each OPE wavelength.

All spectroscopic characterizations were performed using 10 × 10 × 45 mm quartz cuvettes.

### Structural Characterization

The UV circular dichroism (CD) spectra of native proteins, free PDs, and the corresponding PDs–proteins assemblies were measured on a Jasco J-1500 spectropolarimeter (JascoInc, USA) equipped with a Jasco Peltier-type temperature controller (CDF-426S/15), following the experimental protocol described in the paper of Greenfield.^31^ Prior to the measurements, the sample chamber was deoxygenated with dry nitrogen. These conditions were maintained during the experiment. Each CD spectrum was averaged over five accumulations. The as-obtained spectral data were further scrutinized using the K2D3 tool and the database of theoretical spectra provided by DichroCalc according to the procedure described by Louis-Jeune et al.^32^

Attenuated total reflectance Fourier transform infrared (ATR-FTIR) spectra of each sample prepared in Milli-Q or heavy water in the middle infrared range (MIR: 4000–400 cm^–1^) were obtained on a VERTEX 70v vacuum FTIR spectrometer (Bruker FM Technology). ATR-FTIR spectra of albumins in bioconjugates were first corrected to remove pure PDs’ and solvents’ signals, then the fingerprint region of proteins was analyzed using the Gaussian deconvolution method.

Gel permeation chromatography (GPC) experiments were carried out on an ÄKTAexplorer (Amersham Biosciences) system using the Sephadex 75 Increase resin and PBS as proper stationary and mobile phases, respectively. Each retention volume was determined measuring absorbance values at 220, 260, and 280 nm. Prior to the GPC assay, the experimental setup was equilibrated with 50 mM phosphate buffer (pH ∼7.4), and the void volume of a column was estimated using Blue Dextran as a marker (molecular weight (MW) ∼2000 kDa). To establish the calibration curve, seven standard proteins with the MW values ranging from 1.4 to 660 kDa were transferred through the stationary phase with a flow rate of 0.5 mL/min, and their retention volume values were then measured.

Dynamic light scattering (DLS) measurements were performed on a Zetasizer Nano setup (Malvern Instruments) with 633 nm excitation light. The raw data were analyzed using 6.10 software (Malvern Instruments, UK).

The one-dimensional (^1^H and ^13^C) and the two-dimensional heteronuclear single–quantum correlation (HSQC) nuclear magnetic resonance (NMR) spectra of pure PDs were acquired on a Bruker AvanceTM 600 MHz spectrometer. Both **C1K** and **C1Na** were dispersed in deuterium oxide to reach the concentration of 10 mg/mL. The as-measured raw data were analyzed using MestReNova software.

Changes in the pH value were monitored using a Mettler Toledo instrument (SevenCompact Series).

All structural characterizations were performed at room temperature.

### Cell Culture

Bone-marrow-derived macrophages (BMDMs) were obtained from primary bone marrow cells isolated from wild type mice (BEI Resources). The cells were maintained in DMEM supplemented with 10% FBS, 3% l-glutamine, and antibiotics (penicillin and streptomycin). BMDM cells were grown under standard conditions in a humidified incubator at 37 °C in an atmosphere of 95% air and 5% CO_2_. To harvest the adherent cells from confluent cultures, 0.05% trypsin–ethylenediaminetetraacetic acid solution was used, and the cells were centrifuged at 1300 rpm for 5 min.

### Cytotoxicity Tests

Cytotoxicity of free PDs was determined using an MTT colorimetric assay, which is used to measure the cellular metabolic activity as an indicator of cell viability, proliferation, and cytotoxicity. In principle, this assay relies on the reduction of a yellow tetrazolium salt (i.e., MTT) to purple formazan crystals by metabolically active cells.^33^ BMDM cells were seeded onto a 96-well plate (1 × 10^4^/well) and incubated overnight in a 10% FBS complete medium (5%:95% CO_2_/air) at 37 °C. Afterward, a fresh medium was added, and these cells were stimulated for 24 h with varying concentrations of PDs in aqueous suspensions: 1, 10, and 50 μg/mL or LPS (2, 1, 0.5 μg/mL). Subsequently, the supernatant was removed, and the BMDM cells were incubated with the MTT reagent (3 mg/mL) at 37 °C for 4 h. Finally, 100 μL of DMSO was added onto the plate to dissolve the formed formazan crystals. To examine the cells’ response evoked by the PDs, absorbance values of all samples were then acquired on an EnSpire 2300 microplate reader (CLARIO Star microplate reader, BMG Labtech, UK) at 570 nm. The cell viability was expressed as a percentage of BMDM cells relative to the control sample (100%), described as unstimulated cells. As the positive control, the BMDM cells were stimulated with LPS instead of PDs. Statistical analysis was performed using GraphPad Prism 9.1.0 software. The as-obtained results were examined following a one-sample *t*-test. The value of *p* ≤ 0.05 was considered statistically significant.

## Results and Discussion

### TPA of Free PDs

**Figure 1:**
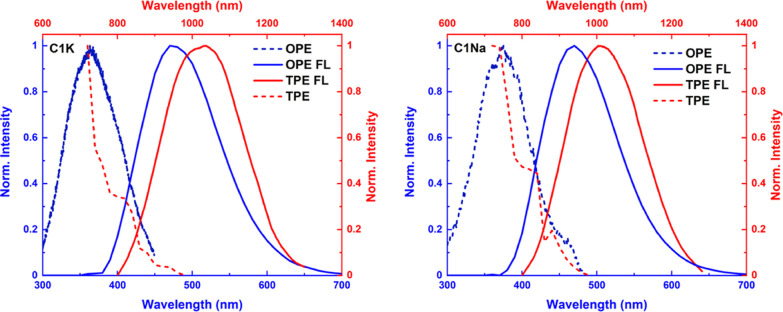
Comparison of normalized one- and two-photon optical properties of **C1K** (left) and **C1Na** (right): OPE (blue dashed), OPE FL (blue solid), TPE (red dashed), and TPE FL (red solid lines) spectra. OPE, OPE FL, and TPE FL correspond to the blue *x*-axis, while TPE is assigned to the red *x*-axis.

In the first step, we investigated the nonlinear absorption properties of pristine PDs (we call them “free PDs”). The multi-photon excited emission spectra were measured in a wide excitation wavelength range (720–1000 nm) of the femtosecond laser system. Strong, green fluorescence was observed under such conditions (Figure 1 and Figures S4 and S7 in the Supporting Information). The most efficient laser excitation is in the range of the first biological optical window,^29^ between 720 and 850 nm.

Double logarithmic plots of the fluorescence signal as a function of incident laser power (log *I*_TPE FL_ vs log *P*_laser_) at 760 nm show quadratic relationships, indicating a predominant role of the TPA mechanism, leading to two-photon excited fluorescence (TPE FL) (Figure S6).

It should be noted that the bright green emission spectra are excitation-dependent. The most intense peaks, centered at 505 nm (**C1Na**) and 507 nm (**C1K**), are observed upon excitation at 720 nm (Figure S5). By moving the excitation wavelength from 720 to 1000 nm, it is possible to shift the TPE fluorescence maxima from 505 to 552 nm for **C1Na** and from 507 to 555 nm for **C1K**. These optical features can indicate the presence of different fluorophore centers, assembled in a single PD.^30,34−37^ In our case, as the PDs are stabilized by the supramolecular and covalent cross-linking (improving the rigidity of nanostructures), the strong multi-photon excited fluorescence may be due to the cross-link enhancement effect (CEE).^30^ We observed a similar spectral behavior for OPE fluorescence of our PDs; however, the TPE emission is slightly red-shifted as compared to that of the corresponding OPE analogue.^30^ Similar trends were reported for protein assemblies (i.e., amyloid fibrils).^38^ In contrast, organometallic supramolecular assemblies and molecular cocrystals possess the constant positions of their OPE and TPE emission peaks.^39−43^ The TPE bands of the PDs are also located at wavelengths shorter than the doubled wavelength maxima of the OPE spectrum (Figure 1), in agreement with the previous nonlinear optical (NLO) studies on carbon dots.^44−46^

To quantify the TPA process, the TPA cross-section (σ_TPA_) values were determined using fluorescein in the basic medium (pH = 11, see Table S2) as a standard, following the formula given below.^47,48^
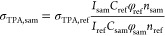
where σ_TPA_ indicates TPA cross-sections [GM]; *I* is the integrated TPE fluorescence intensity (a.u.); *n* is the refractive index of the solvent; and *C* and φ denote the molar concentration and FQY, respectively. Subscripts sam and ref indicate the sample and reference sample, respectively.

**Figure 2:**
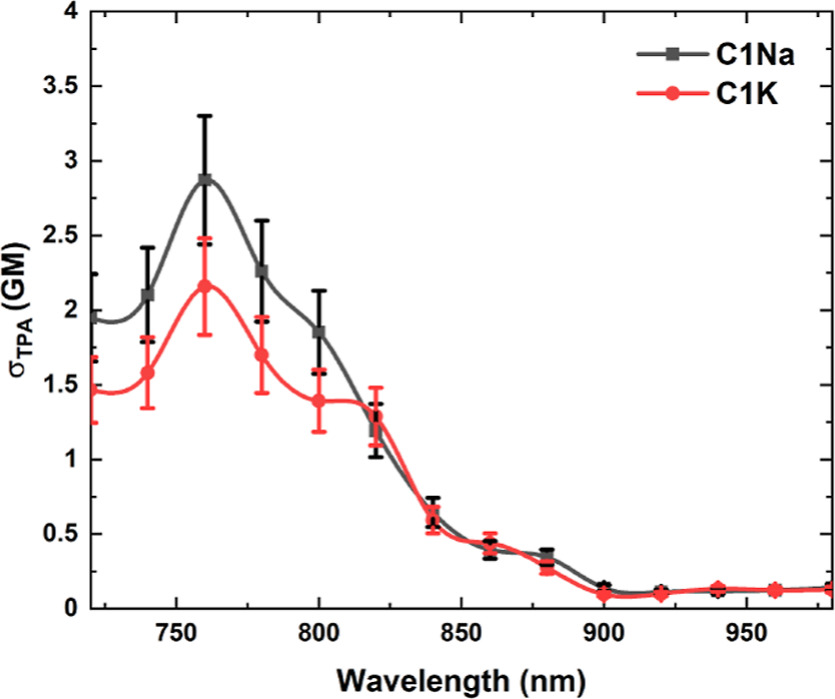
TPA spectra of free PDs in an aqueous medium (pH ∼7.4).

Both types of pure PDs have broad multi-peak TPA spectra and similar σ_TPA_ values, (i.e., at 760 nm, σ_TPA_ = 2.2 GM for **C1Na** and σ_TPA_ = 2.9 GM for **C1K**, Figure 2). The as-obtained TPA results are slightly weaker as compared to the TPA activity of polymeric nanostructures prepared by hydrothermal synthesis (20 GM).^44^ As expected, all PDs exhibit weaker TPA properties than highly ordered carbon dots^49^ and nanographenes (130 GM)^50^ due to the absence of large, π-conjugated structures—one of the most crucial factors determining the TPA of dyes and nanoobjects.^51^ These σ_TPA_ values of PDs are much lower than the σ_TPA_ values of carbon dots reported in the literature, which ranged around 10^3^–10^4^ GM.^46,49,52−54^ However, we note that most of the papers reporting large σ_TPA_ parameters obtained from the TPE FL method provide little details concerning the molarity of fluorophores and the employed methodology. It should be remembered here that carbon dots vary strongly in their designs, from the self-assembled polymeric chains to the highly ordered graphitic structures; the calculations of σ_TPA_ from the TPE FL have to take into account these structural features or, at least, an appropriate estimation of their molar mass (*M*). In our case, the *M* values of PDs were determined by means of the comparative size exclusion chromatography–gel permeation chromatography (SEC–GPC) approach with respect to globular proteins. This method is relevant for diverse carbon dots, including graphene quantum dots^55,56^ and PDs.^57^ The estimated values of MWs are 2.1 kDa (**C1K**), and 2.2 kDa (**C1Na**) (Table S1). These values were used to estimate the molar concentrations and σ_TPA_ parameters; however, it should be remembered that the results from the SEC–GPC experiments approximate the order of *M* values rather than show their exact values.

A better way to quantify the TPA performance of diverse nanostructures with the different chemical character is to scale the σ_TPA_ parameters using a structure-related factor, such as *M* or the species volume.^48,58−60^ The peak σ_TPA_/*M* values have been estimated to be 1.05 × 10^–3^ and 1.32 × 10^–3^ GM mol/g at 760 nm for **C1K** and **C1Na**, respectively. Although these values are lower than those observed for the best TPA dyes or semiconductor nanoparticles (typically 0.1–1 GM mol/g), they still are high enough to consider the TPA-based applications of our PDs.

The major advantage of carbon dots’ is their significant photostability as compared to that of organic fluorescent dyes and inorganic semiconductor nanomaterials. However, in the previous studies, those carbon nanomaterials were exposed to the UV or green lights only.^61,62^ To evaluate the possibility of applying the PDs for bioapplications involving the illumination in the first biological window, we monitored their photostability upon exposure to strong laser irradiation (at 760 nm) using UV–vis and conventional fluorescence spectroscopy techniques. The PDs do not show evident photobleaching for up to 30 min of exposure to the laser beam (Figure S8).

### Cytotoxicity Assays

**Figure 3:**
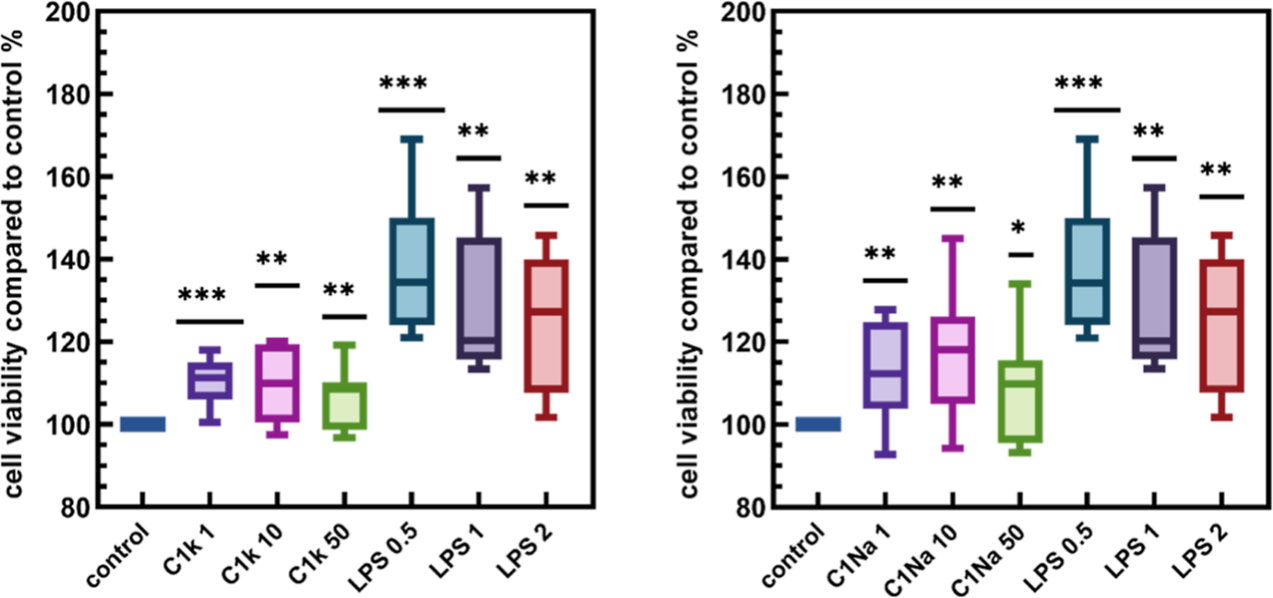
Cytotoxic effect of PDs on BMDM cells viability using an MTT assay. The cells were treated with PDs at varying concentrations: 1, 10, and 50 μg/mL. The positive control was cells stimulated with LPS (2, 1, 0.5 μg/mL). The negative control was untreated cells. It was shown that PDs did not affect BMDM cell viability after 24 h of incubation. Results are presented as mean to min–max based on three independent experiments. One sample *t*-test was used to examine the mean differences between samples and the control; (*) *p* ≤ 0.05; (**) *p* ≤ 0.001; and (***) *p* ≤ 0.0001 vs control.

The cytotoxicity of free PDs was tested in vitro on the BMDM cell line. The BMDM cells were exposed for 24 h to three concentrations of the PDs: 1, 10, and 50 μg/mL, and the cell viabilities were estimated using the MTT assay. For these concentrations, in the presence of PDs, we observed an increase in the growth of BMDM cells (by ca. 10% compared to that of the control sample) upon the incubation process (Figure 3). This observation is contrary to previously reported results suggesting that the cell viabilities decrease in the presence of diverse carbon dots, even at relatively low nanomaterial concentrations (e.g., 5–50 μg/mL);^63−68^ this decrease becomes sharp when the quantity of carbon dots in the system increases.^67^ Liu et al. also demonstrated the photodegradation-induced cytotoxicity of carbon dots exposed to white light irradiation.^69^ Hence, the absence of toxic effects of the currently studied PDs on BMDM cells and the outstanding photostability may constitute their unique properties; this hypothesis needs detailed verification.

### Enhancement of One-Photon Excited Emission

**Figure 4:**
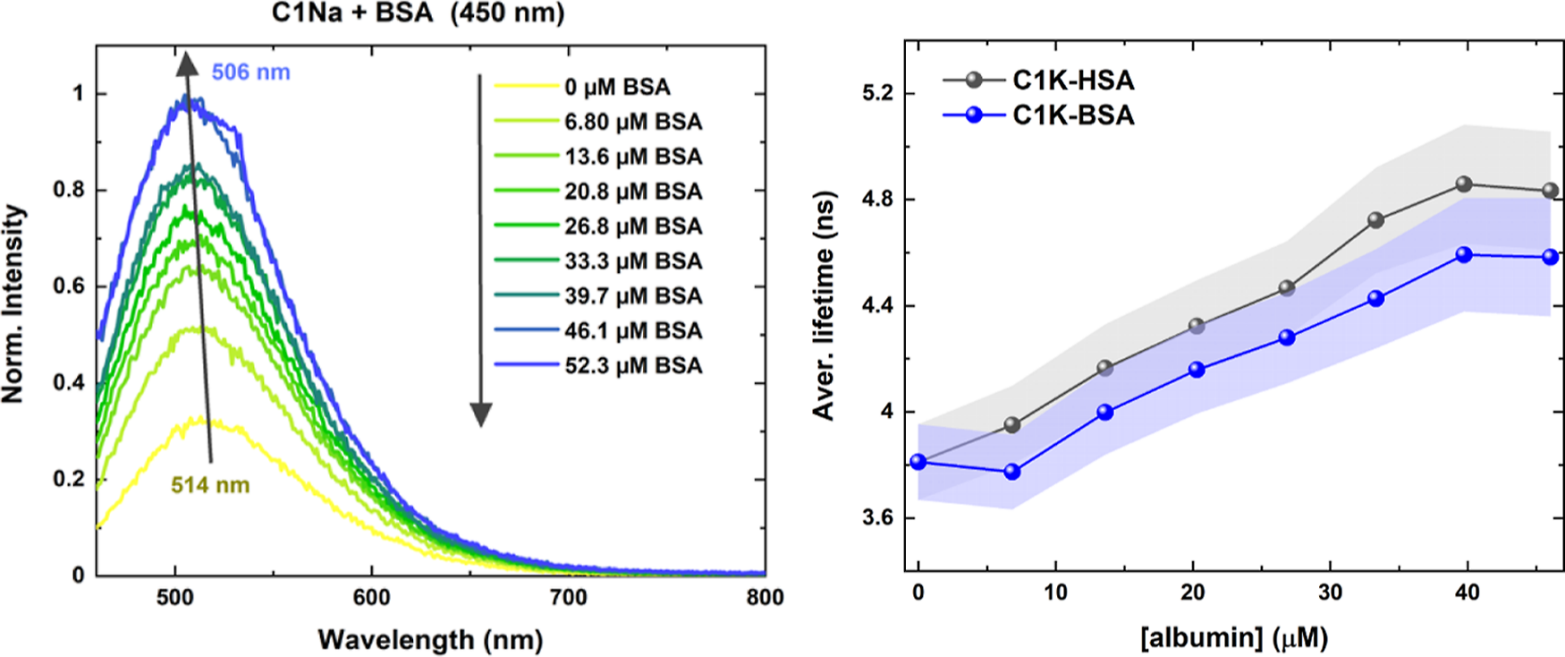
OPE fluorescence of **C1Na** in the presence of BSA, excited at 450 nm (left). Evolution of the average fluorescence lifetime in the proteinaceous environment (right). Gray and blue areas correspond to the error bars.

To evaluate the influence of albumins on the optical features of PDs, extinction and fluorescence spectra of protein-titrated **C1Na** and **C1K** at three simulated physiological conditions were recorded. Figures 4 and S9–S11 show the evolution of fluorescence spectra when the albumin concentrations increase. For all samples (**C1K/BSA**, **C1K/HSA**, **C1Na/BSA**, and **C1Na/HSA),** the fluorescence intensity increases with the increasing proteins concentrations. In the aqueous media, upon illumination at 350, 377, and 450 nm, a hypsochromic shift (Δλ) of the spectra is observed. The excitation at these wavelengths do not induce a direct excitation of proteins, only weak emission signals at 434, 449, and 505 nm (Figures S12 and S13 and Table S3). The protein-induced Δλ and the OPE fluorescence enhancement coefficient (FEC) are dependent on the excitation wavelength (Table S4). The FEC is the highest in the presence of HSA, even reaching 276% [**C1Na-HSA**; λ_exc._ = 450 nm and Δλ ∈ (2; 20) nm]; the FQYs of PDs also increase in the presence of proteins (Table S4). A similar observation was made for each aqueous sample at given physical conditions, suggesting that PDs–albumins interactions are not sensitive to the chemical composition of aqueous media. This fact pleads in favor of PDs being used for further studies in complex biological environments. More detailed results are listed in the Supporting Information.

We also measured the evolution of fluorescence decays of PDs upon binding proteins using the TCSPC method (λ_em._ = 471 nm and λ_exc._ = 377 nm). The decay profiles were fitted using the tri-exponential equation, resulting in three fluorescence lifetime components (see the Supporting Information). We found that the coupling of blood proteins to PDs slows down the decays. Figures 4, S18, and S19 show an increase in the average fluorescence lifetime (by 1 ns with respect to that of unbound PDs). Such elongated fluorescence lifetimes along with higher FQYs (Table S6) indicate a decreasing contribution of non-radiative relaxation pathways: the presence of albumins seems to prevent different molecular motions of PDs’ sub-structures and reduce dynamic fluorophore–solvent interactions,^70^ also suggesting the high importance of modifications of the fluorophores’ environment.^71^ Similar observations were already reported in the literature; the adsorption of proteins onto the surface of gold and silver nanoclusters also led to longer fluorescence lifetimes.^72,73^

### Enhancement of Two-Photon Excited Emission

Although the albumins do not show any detectable TPE emission,^74^ the TPE fluorescence of PDs is significantly enhanced in their presence. The fluorescence intensity increases with the concentration of proteins (Figures S14 and S15), which also induces the hypsochromic shift (up to Δλ ∼17 nm). As for OPE fluorescence, the enhancement parameters are sample composition- and excitation-dependent (Table S5); the highest FEC values are again observed for the **C1Na-HSA** sample (λ_exc._ = 850 nm). The protein-induced enhanced TPE FL showed outstanding photostability upon exposition to the femtosecond laser irradiation. The fluorescence dependence on excitation power (log *I*_TPE FL_ vs log *P*_laser_) is quadratic (Figure S16), as expected for the TPE mechanism.

It is worth noting that albumin-enriched PDs exhibit stronger TPA properties than their free analogues. The maximum σ_TPA_/*M* values are higher than those calculated for pristine PDs: 2.9 × 10^–3^ GM mol/g (for **C1K-HSA**), 2.0 × 10^–3^ GM mol/g (for **C1Na-HSA**), and 2.2 × 10^–3^ GM mol/g (for **C1K-BSA**) at 760 nm. The σ_TPA_/*M* values of **C1Na-BSA** remain unchanged (1.3 × 10^–3^ GM mol/g at 760 nm).

**Figure 5:**
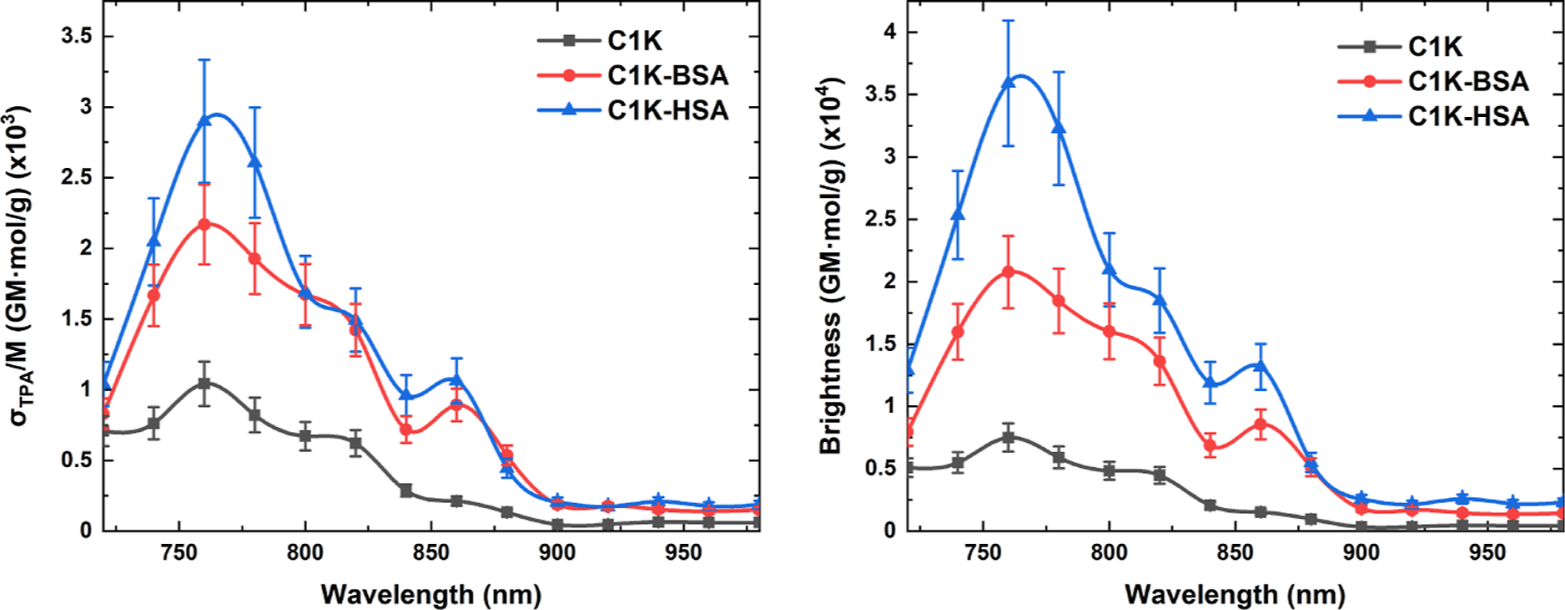
Molar-weight-scaled TPA cross-section spectra of free and protein-including **C1K** systems (left) and the corresponding two-photon brightness spectra (right).

These fundamental TPA parameters are not sufficient to compare the performance of albumin-containing mixtures with that of free PDs in terms of TPE fluorescence applications. To quantify the evolution of the albumin-sensitive TPA activities of PDs that can lead to the emission process, we determined their two-photon brightness (φ·σ_TPA_/*M*). If we combine the increased FQYs of PDs dispersed in the proteinaceous environment with varying TPA performances (σ_TPA_/*M*), we can confirm a significant improvement of TPA properties for each albumin–PDs composition. It should be noticed that the proteins differ in the enhancement efficiency: HSA-including samples provide 2.8–4.8-fold higher σ_TPA_/*M* values than BSA-including systems (1.4–2.8-fold enhancement in the whole wavelength range). The strong increase of the TPA cross-sections normalized by molar mass and presented as two-photon brightness values are comparatively illustrated in Figures 5 and S17. The data reported so far in the literature showed that albumins reduce (e.g., 6.1-fold decrease)^75^ or, at the best, enable an unchanged two-photon brightness of fluorescent probes.^16^ Therefore, the present result showing the above-described enhancement of the fluorescence response of the PDs in the presence of albumins is unique and promising. To the best of our knowledge, this is also the first report demonstrating such a behavior in fluorescent carbon nanomaterials.

### Structural Characterization

**Figure 6:**
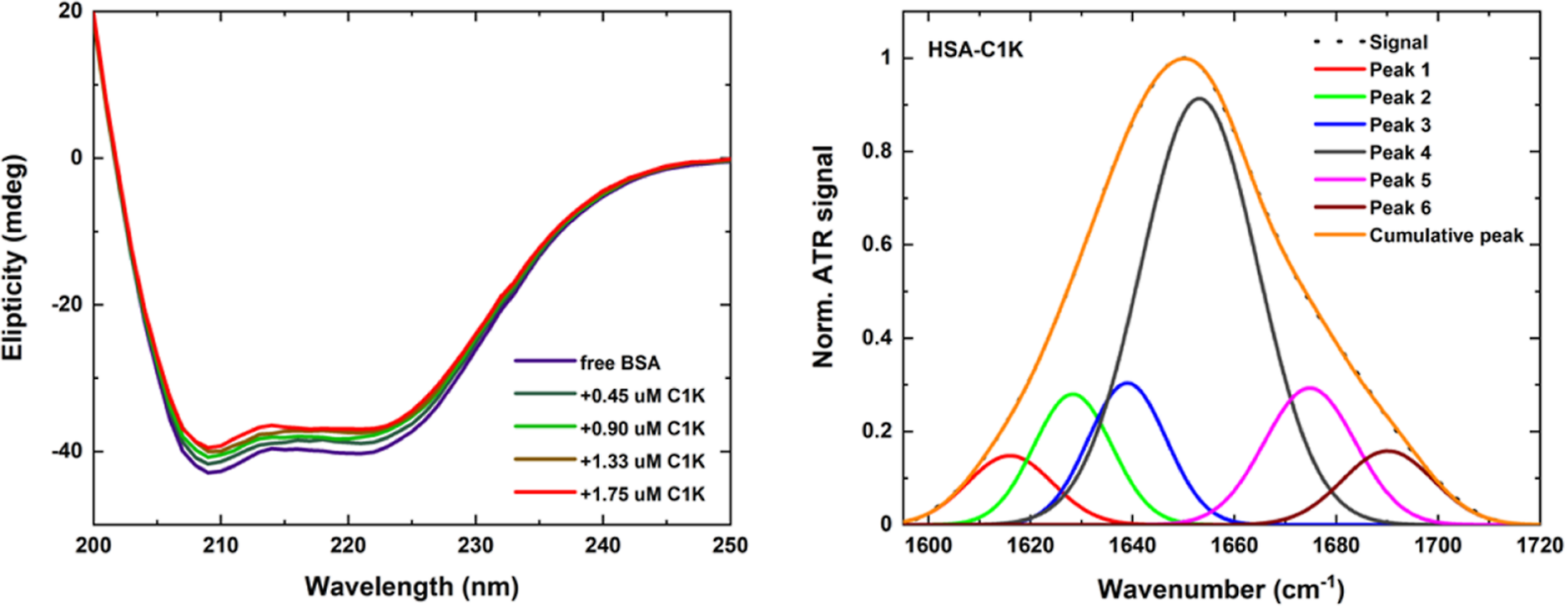
UV CD spectra of BSA doped with **C1K** (left). Gaussian deconvolution of the amide I band of HSA in the **C1K**-including composites (right).

The PD-induced conformational changes occurring within the albumins (i.e., the modification of secondary and tertiary protein structures) were studied using UV CD and FTIR spectroscopy techniques. Following the procedure provided by Louis-Jeune et al.,^32^ the structural studies reveal that the CD spectra of native blood proteins are composed of two negative ellipticity peaks at 209 and 222 nm and one embedded minor component at 218 nm (Figures 6 and S19). These spectral features indicate the predominant role of the α-helix relative to that of β-sheets and unordered components (64 and 57% for HSA and BSA, respectively), a result that is consistent with the literature data.^12,16,25,76^ Pure PDs, on the other hand, show no chiroptical effects in a wide wavelength range. Upon the titration of the proteins with PD colloidal solutions, the ellipticity values at 209 and 222 nm tend to slightly decrease. Such CD changes are attributed to the rearrangement of the secondary protein structure of albumins: the α-helix content decreases and random coil components starts to increase. However, the presence of both **C1Na** and **C1K** has only a minor effect on the secondary protein structure, resulting in weak protein unfolding: the α-helix contents are reduced (by ca. 4% in HSA and 5% in BSA), while the β-sheet contents remain constant (Figure S21). This confirms that our PDs interact with the proteins without denaturing them.

To identify any conformational changes of proteins, the ATR FTIR spectra of pure PDs, free blood plasma proteins, and PDs–albumin mixtures were recorded. According to the literature, BSA and HSA plasma proteins display nine characteristic absorption peaks (i.e., amide bands A–B and I–VII), which are assigned to different vibrations from N–H, C=O, and C–N bonds.^77,78^ The major absorption signals correspond to the amide I band (C=O stretching) and amide II band (C–N stretching and N–H bending). They are located in the range of 1500–1750 cm^–1^, considered as the fingerprint region of proteins and usually studied in the quantitative conformational analysis.^38,77−82^ All normalized amide I bands of proteins–PDs samples exhibit only a small broadening and a blue shift. Amide II bands decrease slightly in the presence of varying contents of PDs (see Figure S22). This implies negligible changes in the tertiary and secondary protein structures of albumins. The Gaussian deconvolution of the amide I band uncovers five peaks in the case of native proteins and six components upon PDs’ binding (Figures 6 and S23). The position of the main absorption band is nearly constant (1652.5–1654.5 cm^–1^), and its intensity decreases insignificantly for bound proteins (as compared with that of free albumins), thereby indicating an almost unchanged contribution from α-helix domains (BSA: from 60.1 to 59.4% and HSA: from 64.9 to 59.0%). Smaller absorption peaks, centered at 1621 cm^–1^ (BSA: 1623.5 cm^–1^), 1637 cm^–1^ (BSA: 1636 cm^–1^), 1679 cm^–1^ (BSA: 1674 cm^–1^), and 1691 cm^–1^ (BSA: 1688 cm^–1^), indicate the presence of β-sheets, random coils, β-turns, and β-antiparallel sheets. Their amplitudes and positions are only slightly affected by the PDs (Table S7). All four mixtures also show new minor absorption peaks at 1611 cm^–1^ (**C1Na-BSA**) or 1615 cm^–1^ (**C1Na-HSA**, **C1K-HSA**, and **C1K-BSA**), which indicate the formation of the intermolecular β-sheets (2.2–5.1%) and, consequently, imply negligible proteins’ aggregation.^83−85^ These results confirm that the binding between globular proteins and PDs do not induce albumin unfolding and are in a good agreement with the CD data.

To monitor the aggregation processes for PDs or proteins and the potential formation of their bioconjugates, the SEC–GPC method was also used. As depicted in Figure S24, blood plasma proteins exhibit the narrow major peaks at the elution time of their monomers at ca. 18.3 min and a smaller one for their dimers at 16.2 min with no significant shifts in the presence of PDs. Similarly, chromatograms of PDs with serum albumins display only a minor shift (less than 0.8 min) with respect to that of free PD samples.

The abilities of albumins to form aggregates and bioconjugates were also verified using the DLS method. It is worth noting that similar tendencies were found for hydrodynamic diameters (*D*_hydr_) of albumins in native forms and in the presence of PDs (Table S8, ∼0.3 nm of variations). These results indicate that the proteins do not form any bigger conjugates with PDs or with themselves.

Altogether, the investigations prove that, in contrast to several previous studies on albumin nanostructure assemblies that showed significant protein unfolding,^23,25,86^ the structural properties of albumin–PD mixtures are preserved. It should be remembered that any structural rearrangements of proteins may lead to the loss of their biological activity. We conclude then that our PDs have good biocompatibility toward the blood proteins.

Furthermore, since mixing PDs and albumins does not noticeably change their MW and diameter values, we can deduce that protein–marker conjugates are not formed.^8,22^ Formation of aggregates with the aggregation-induced emission^15^ reported previously is not observed here.

### Temperature Effect on PDs and Their Optical Activity

To correctly evaluate the real potential of PDs in albumin sensing and probing, it is essential to explore their properties in the thermodynamic conditions that mimic the actual conditions occurring in biological samples. Two aspects have to be considered: (i) the thermal stability of PDs and (ii) the temperature effect on the OPE emission activity of PD–albumin conjugates.

**Figure 7:**
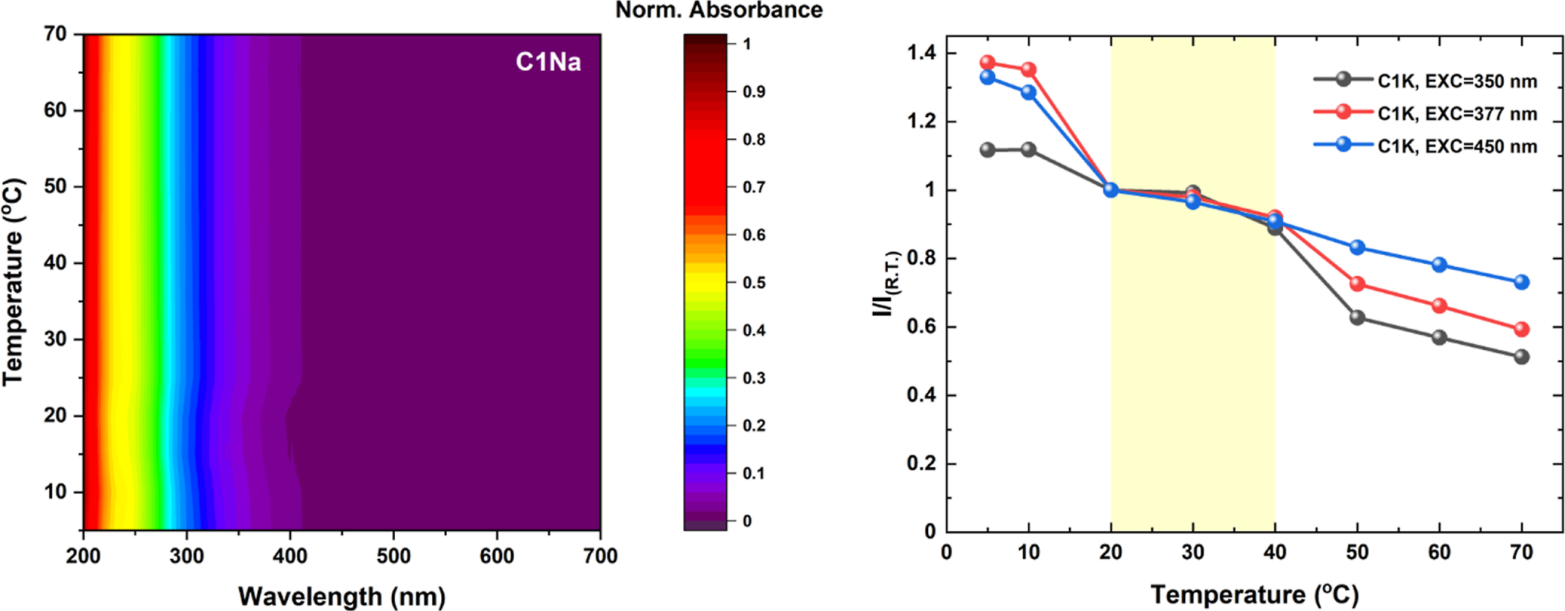
Normalized temperature-dependent extinction map of **C1Na** (left) and the evolution of the integrated emission intensity of **C1K**, scaled by the fluorescence intensity observed at 20 °C vs temperature values (right).

To study the thermostability of both **C1K** and **C1Na** in aqueous suspensions, the extinction spectra of PDs at varying temperatures were acquired first. The profiles of UV–vis extinction spectra of PDs remain unchanged in the temperature range from 5 to 70 °C (Figures 7 and S25). Moreover, small absorbance changes at the absorption band at 240 nm (π–π* transitions) and a negligible absorption peak of PDs at 310 nm (*n*–π* transitions) are observed in the whole temperature range. These findings prove that in the analyzed temperature range, single PDs are thermodynamically stable: neither undesirable aggregation nor degradation processes occur.

To explore the influence of temperature on the fluorescence of PDs and the albumin conjugates (**C1K-BSA** and **C1K-HSA**), the emission spectra was recorded at temperatures varying from 5 to 70 °C. The integrated fluorescence intensities (*I*) were then normalized by the corresponding integrated intensity measured at 20 °C (*I*_R.T_).

Figures 7, S26, and S27 show the variation of OPE emission spectra of PDs and PD–albumin samples as a function of temperature. The fluorescence intensity decreases with increasing temperature, but no spectral shifts are observed. The emission, the most intense at a low temperature (below 20 °C), gradually decreases above 40 °C. This variation may result from the different contribution of non-radiative relaxation pathways of PDs in an aqueous environment. From a structural point of view, rapid motions of solvent molecules and nanostructures at higher temperatures^87^ causes weakening of their hydrogen bonds.^88^ In contrast, at low temperatures, immobilization of PDs is stronger. A similar temperature-dependent emission intensity of carbon dots was reported by Yu et al.^89^ Both PDs show high and relatively constant OPE fluorescence between 20 and 40 °C (almost 90% of the *I*_R.T._ value at λ_exc._ = 377 nm, *T* = 40 °C for **C1K**).

Similar stability trends were also observed for the PD–albumin systems, indicating that a significant temperature resistance remains unchanged in the presence of proteins. It confirms the high potential of PDs to be applied in both in vivo and in vitro biochemical assays.

### pH Dependence of PD Fluorescence Properties

**Figure 8:**
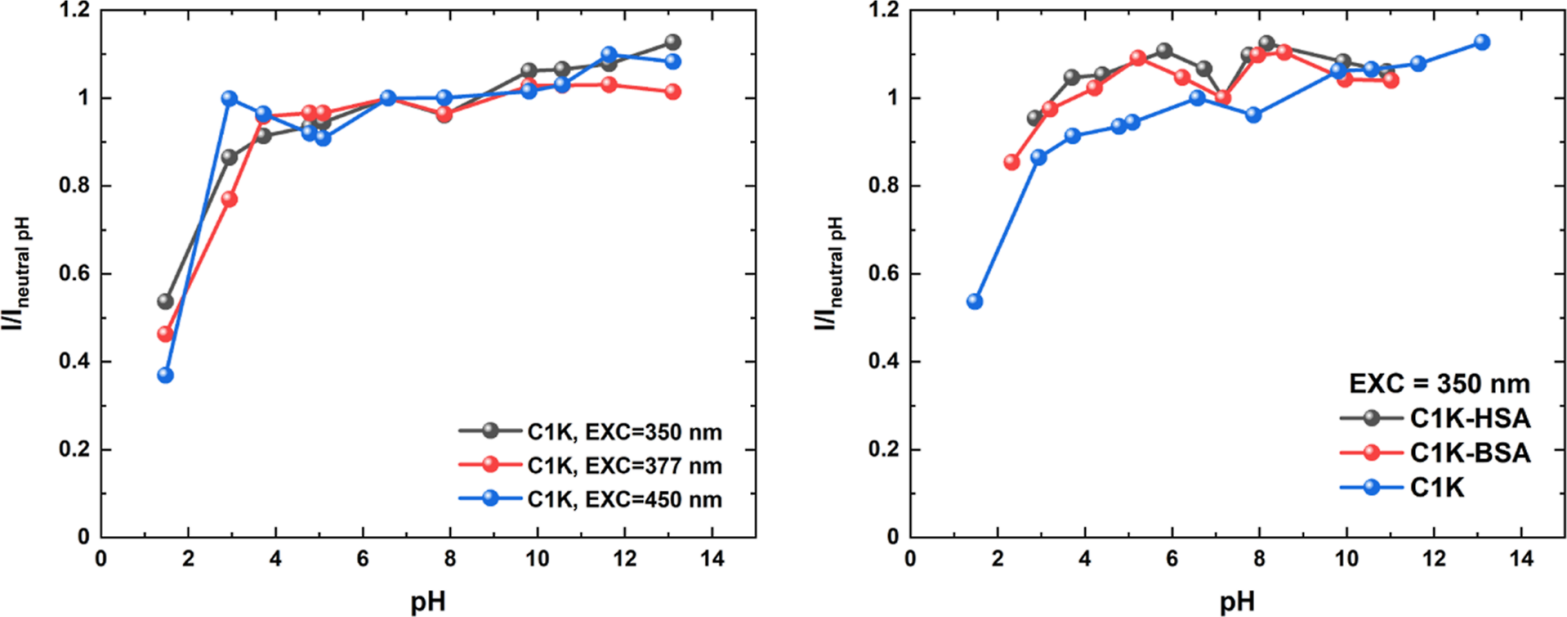
Evolution of the integrated fluorescence intensity vs pH values for **C1K** (left) and comparative analysis for **C1K**, **C1K-BSA**, and **C1K-HSA** (right). The integrated fluorescence intensities (*I*) were scaled by the relevant reference value at neutral conditions (*I*_neutral pH_).

The OPE emission spectra of PDs and PD–albumin systems were monitored taking into account the following factors: (i) pH values, (ii) molecular interferents, and (iii) other proteins. The pH constitutes the essential parameter in biochemical assays (for example in biosensing). To explore how the pH of an aqueous environment affects the fluorescence activity of PDs and their interactions with albumins, the pH of solutions was tuned using sodium hydroxide and hydrochloric acid solutions (1 M), then OPE emission spectra were recorded at three excitation wavelengths (Figures 8 and S28), Both PDs exhibit pH-responsive emission characteristics but differ slightly in their behavior. The fluorescence of free **C1K** remains almost unchanged in the wide pH range (2.9–13.1); a sharp drop is observed only in the acidic conditions. For **C1Na**, the emission intensity first gradually increases from *I*/*I*_neutral pH_ ∼0.60 (for pH = 2.9) to ∼1 (for pH = 6.7), reaches a plateau in neutral (physiological) conditions, and then grows. Since both PDs are rich in polar groups (Figures S31–S34),^30,90,91^ their structure may account for a pH-responsive OPE fluorescence. More specifically, carboxyl groups can be deprotonated when pH is greater than 2.9, thereby forming gradually the negatively charged “protective shell” for single PDs. The negative charge also originates from the present enol conjugates. Such negative charge sites seem to account mostly for the OPE fluorescence stability in the wide pH range.^92^ As expected, the lowering of pH below 2.9 leads to the protonation process of carboxyl groups and, as a result, reduced emission intensity.^92,93^ Note that the numerous hydroxyl moieties also play an essential role; they form the strong hydrogen bonding network and enol conjugates. Additionally, at an alkaline pH, hydroxyl groups undergo progressive deprotonation, resulting in a stronger emission signal.^30^

The OPE emission of free PDs remains relatively stable at varying pH conditions; the proteinaceous environment can even enhance this property. According to Figure 8, the *I*/*I*_neutral pH_ values for **C1K** remain unchanged upon the protein binding, at both acidic and alkalic conditions (2.0 < pH < 11.0). As the albumins are positively charged at pH > 4.7 (isoelectric point: pI_BSA_ = 4.7),^94^ it is evident that the electrostatic forces are important between PDs and albumins. Moreover, the strong hydrogen bonding should be also considered—globular proteins and PDs have common potential donor (i.e., hydroxyl or amine) and acceptor (carbonyl) groups.

We conclude that serum albumins act as remarkable stabilizing agents for PDs, protecting them against the excess of H^+^ and OH^–^ ions and reducing the protonation/deprotonation processes.

### Molecular Interferents versus OPE Fluorescence

**Figure 9:**
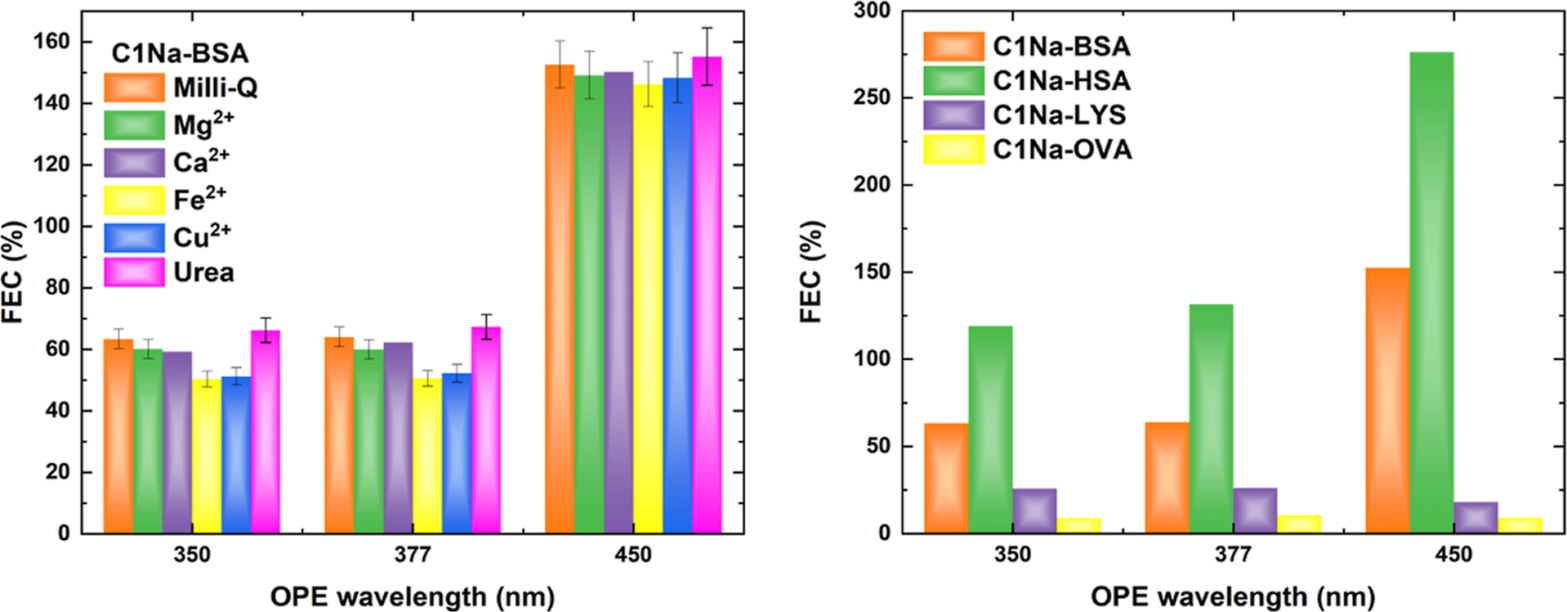
Interferents’ effect on the FEC values for C1Na-BSA (left) and selectivity of C1Na toward various proteins (right).

Blood plasma constitutes a complex biochemical system containing not only globular proteins but also a variety of other biological and chemical species, for instance, simple organic molecules (e.g. glucose, uric acid, and urea) and ionic components.^95,96^ Therefore, we analyzed the OPE fluorescence signals of PD–albumin samples exposed to representative blood plasma components, such as urea molecules (50 mM) and metal ions (50 mM Mg^2+^, 50 mM Ca^2+^, 10 mM Fe^2+^, and 50 mM Cu^2+^). No significant changes in FEC values were found in the presence of alkaline earth metal cations. Both transition metal ions reduce the fluorescence enhancement efficiency only slightly. The presence of urea improves the FEC parameter (Figures 9 and S29). Overall, the variations of FEC in the presence of common interfering agents are small, indicating their minor influence on the PD–albumin system. Therefore, these findings offer the advantage of PDs for sensing applications in biological samples.

### Selectivity toward Proteins versus Fluorescence Enhancement

Since the fluorescence signal of PDs is enhanced in the presence of BSA and HSA, it is crucial to examine their emission in the presence of other common proteins, for instance, LYS and OVA. These proteins differ from BSA and HSA in molecular weights (MW_LYS_ = 14 400 Da and MW_OVA_ = 45 000 Da)^97^ and charge under neutral conditions (pI_LYS_ = 10.7 and pI_OVA_ = 4.5).^97,98^Figures 9 and S30 clearly show that all four types of proteins are able to improve OPE emission signals of PDs. However, the enhancement efficiency depends strictly on protein: the FEC parameter is 26.1% for **C1Na-LYS** (λ_exc._ = 377 nm) and 14.9% for **C1K-OVA** (λ_exc._ = 350 nm). These values are several folds weaker than serum albumins, allowing a facile discrimination of BSA and HSA.

### Albumin–PD Interaction Mechanism

To gain deep insights into the mechanism of interactions and the origin of the sensitive albumin probing, several observations have to be taken into account.

First, no aggregating conjugates were found using the SEC–GPC and DLS assays, suggesting that both PDs and globular proteins disperse well as free species in aqueous environments. Knowing that both chemicals are similar in size,^99,100^ no specific binding between them occur, as opposed to proteins’ interactions with small molecular species.^2,12,15^

The above condensate-free model is also consistent with the ATR-FTIR and CD spectroscopy results, which excluded the formation of the covalent bonding between PDs and albumins. The interactions between PDs and blood plasma components should be then either electrostatic, van der Waals, hydrogen bonding, or hydrophobic in nature.^101^ Analyzing the internal structure of free PDs and their fluorescence response in varying pH indicates that PDs are rich in different polar sub-units, including hydroxyl, carboxyl, and carbonyl (enol) groups. Their presence accounts for the pH-responsive emission of free PDs where the negative charge sites of PDs favor electrostatic interactions with positively charged albumins under neutral conditions. They also form a strong hydrogen bonding network. In addition, the contribution of hydrophobic forces should be considered—both albumins and PDs possess non-polar structural domains, such as subdomain IIa^2,102^ and aliphatic hydrocarbon chains,^30^ respectively.

The improvement of fluorescence properties of PDs may originate from the substantial modification of their environments. Two scenarios appear to be possible. First, albumins may act as a sponge—they create more hydrophobic conditions, which prevents PDs from water molecules, slowing the relaxation rate and increasing the contribution of radiative pathways.^87^ Second, serum albumins may induce steric effects, which hinder effective rotations of flexible aliphatic sub-units of PDs, as reported for small organic molecules,^71^ resulting in a more rigid architecture with a decreasing role of non-radiative relaxation pathways. Note that the interactions between PDs and albumins are sensitive to protein/PD stoichiometry and are the most pronounced for the protein: a nanomaterial molar ratio of 4:1 (Figure S20).^103^

The explanations given above facilitate the differentiation of enhanced fluorescence phenomena from the fluorescence quenching processes, which lower the fluorescence intensity, relying on either dynamic (effective collisions with quenchers) or static (the formation of the non-fluorescent complex) mechanisms.^70,104^

### Protein Sensing

The strong enhancement of OPE and TPE fluorescence of PDs with almost no morphological alterations of proteins and the absence of cytotoxicity have prompted us to explore the possibility of applying the PDs in the non-destructive and selective recognition of proteins. For this purpose, we performed the OPE fluorescence titration experiments for low concentrations of proteins, that is, in the linear relationship region (Figure S35), and estimated the limit of detection (LOD), defined as.^105,106^
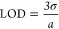
where LOD is the limit of detection (μM or mg/mL), σ denotes the standard error of the regression line, and *a* corresponds to the slope of the linear relation between the integrated fluorescence intensity of PDs and the concentration of albumins.

**Scheme 1:**
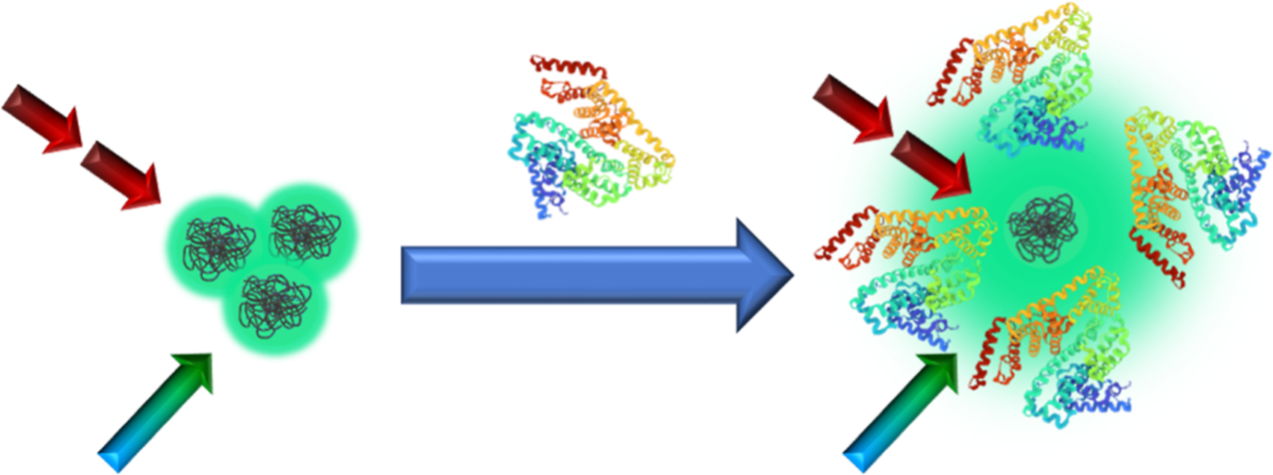
Correlation between the BSA-Sensitive Distribution of PDs and Their One- (Blue-Green Arrow) and Two-Photon (Dark Red Arrows) Excited Fluorescence Response (Green Cloud) The 3D structure of BSA was reprinted from the Protein Data Bank (PDB ID: 3V03), deposited by Majorek et al.

The calculated LOD values for HSA range from 0.21 mg/mL (3.22 μM, **C1K** for excitation at 350 nm) to 0.36 mg/mL (5.50 μM, **C1K** at 450 nm), as listed in Table S8. These values are comparable to other reported LOD values^16^ and suggest that our PDs can be successfully used in the fluorescence enhancement-based recognition of albumins. Their performance in complex biological systems, which include other analytes and potential interfering agents, needs to be analyzed in future studies (Scheme 1).

## Conclusions

Acetone-derived PDs were investigated in terms of their NLO activity, biocompatibility, and use in non-destructive and reversible albumins sensing and probing. The as-prepared PDs revealed intense TPE fluorescence upon excitation in the first biological window and reasonably high TPA cross-sections (up to 2.9 GM at 760 nm). The cytotoxicity assays showed unique proliferation effects in a wide concentration range for both types of PDs. OPE and TPE fluorescence of PDs shows strong sensitivity toward globular albumins, up to 27 times stronger than the fluorescence intensity of free PDs and featuring longer lifetimes. Conformational studies demonstrated that the protein–PD interactions did not affect significantly the secondary protein structure of blood albumins, nor induced aggregates formation. Therefore, the PDs seem to be promising candidates for non-destructive and reversible probing of albumins in various physiological conditions, having great perspectives to be further explored in complex biological samples with a variety of potential chemical interferents and analytes.

## Supporting Information Available

More detailed descriptions of data treatments of CD and FTIR spectra, along with all spectroscopic and chromatographic results (PDF)

## Abbreviations

PDs: polymer dots
OPE: one-photon excitation
TPE: two-photon excitation
TPA: two-photon absorption
OPE FL: one-photon excited fluorescence
TPE FL: two-photon excited fluorescence
NLO: nonlinear optical
PBS: phosphate-buffered saline
BSA: bovine serum albumin
HSA: human serum albumin
LYS: lysozyme
OVA: ovalbumin
DMEM: high-glucose Dulbecco’s modified Eagle’s medium
FBS: fetal bovine serum
LPS: bacterial lipopolysaccharide
MTT: 3-(4,5-dimethylthiazol-2-yl)-2-5-diphenyltetrazolium bromide
TCSPC: time-correlated single-photon counting
CD: circular dichroism
ATR-FTIR: attenuated total reflectance Fourier transform infrared
MID: middle infrared
BMDM: bone-marrow-derived macrophage
SEC: size-exclusion chromatography
GPC: gel permeation chromatography
CEE: cross-link enhanced effect
DLS: dynamic light scattering
