# Supplementary material for: Non-toxic Polymeric Dots with the Strong Protein-Driven Enhancement of One- and Two-Photon Excited Emission for Sensitive and Non-destructive Albumin Sensing
Source: ACS Appl Mater Interfaces. 2022 Aug 26;14(35):40200–13. doi: 10.1021/acsami.2c08858 (PMC9460497; doi:10.1021/acsami.2c08858)
Supplement: Supplementary file 1 — am2c08858_si_001.pdf [file am2c08858_si_001.pdf]

Supporting Information

Non-Toxic Polymeric Dots with the Strong Protein-Driven Enhancement of One- and Two-Photon Excited Emission for Sensitive and Non-Destructive Albumins Sensing

Sebastian G. Mucha,[a] *Marta Piksa,*[b] *Lucyna Firlej,*[a] *Agnieszka Krystyniak,*[c] *Mirosława O. Różycka,*[d] *Wioletta Kazana,*[b] *Krzysztof J. Pawlik,*[b] *Marek Samoć,*[c] *and Katarzyna Matczyszyn*[c*]

[a]Laboratoire Charles Coulomb, UMR5221, Université de Montpellier (CNRS), 34095 Montpellier, France;

[b]Ludwik Hirszfeld Institute of Immunology and Experimental Therapy, Polish Academy of Science, 53-114 Wroclaw, Poland;

[c]Institute of Advanced Materials, Wroclaw University of Science and Technology, 50-370 Wroclaw, Poland;

[d]Department of Biochemistry, Molecular Biology and Biotechnology, Wroclaw University of Science and Technology, 50-370 Wroclaw, Poland;

corresponding author: katarzyna.matczyszyn@pwr.edu.pl

Contents:

PDs' notations.....3
Absorption and fluorescence spectra of serum albumins.....4
Molar mass from gel permeation chromatography.....5
Two-photon excited fluorescence of free PDs.....6
Two-photon absorption cross-section of reference dye.....8
Photobleaching resistance.....9
Enhanced one-photon excited fluorescence of PDs.....10
Linear optical properties of free albumins.....13
Enhanced two-photon excited fluorescence of PDs.....14
The fluorescence enhancement coefficients.....17
Absolute fluorescence quantum yields.....18
Two-photon absorption cross-section and brightness.....19
Fluorescence decays.....20
Stoichiometry.....21
Circular dichroism.....22
Fourier-transform infrared spectra.....24
SEC-GPC experiments.....27
Thermal stability of PDs.....28
Temperature-dependent one-photon excited emission.....29
pH effect on the fluorescence activity.....31
Molecular interferents *vs.* OPE fluorescence.....32
Selectivity towards proteins.....33
NMR characterization of PDs.....34
Albumins' sensing.....38
References:.....40

PDs' notations

Two hydrophilic fractions of acetone-derived PDs are considered in this work:

- **C1Na** denotes PDs received from sodium hydroxide-mediated (NaOH) synthesis;
- **C1K** corresponds to PDs fabricated using potassium hydroxide (KOH) as a catalyst.

More details regarding the synthesis and purification processes along with fundamental characterizations of their structural and linear optical properties.[1]

Absorption and fluorescence spectra of serum albumins

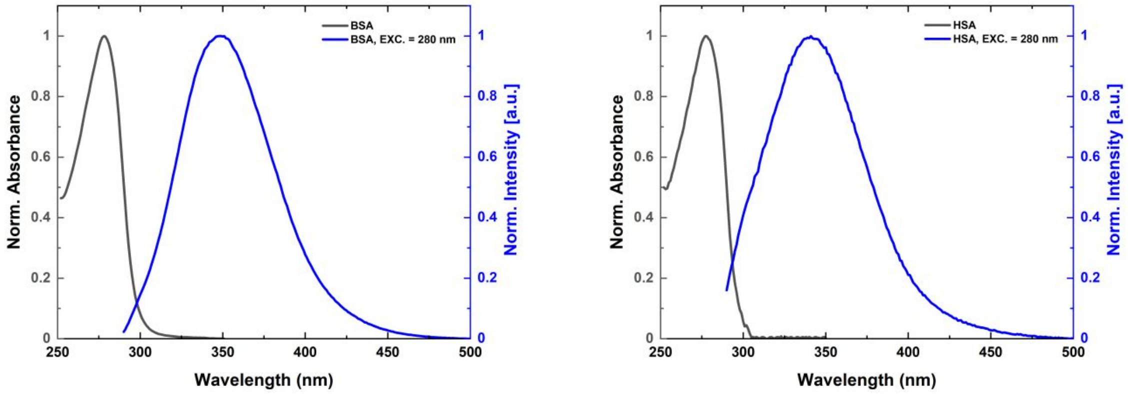

Figure S1. Normalized absorption (black) and fluorescence (blue) spectra of native albumins.

Molar mass from gel permeation chromatography

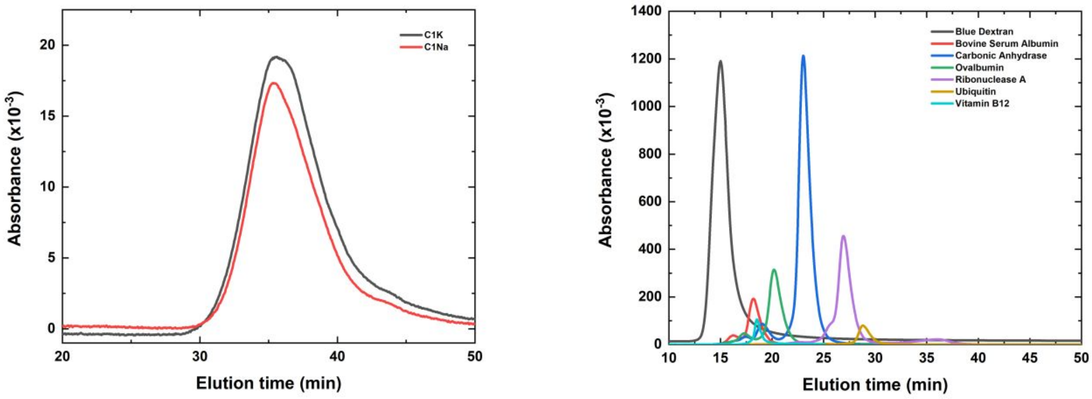

Figure S2. Chromatograms of standard proteins and pure PDs.

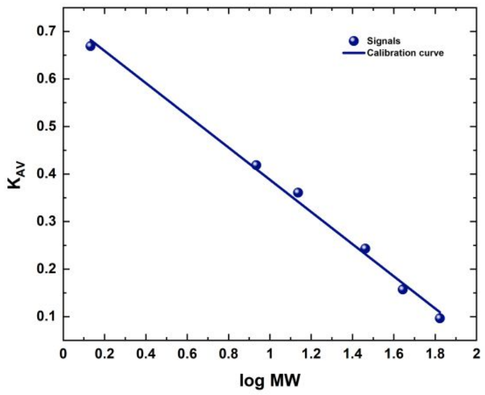

Figure S3. The calibration curve for the calculation of molar masses.

Table S1. The molar mass values of free PDs.

Sample	M (kDa)
**C1K**	2.07
**C1Na**	2.18

Two-photon excited fluorescence of free PDs

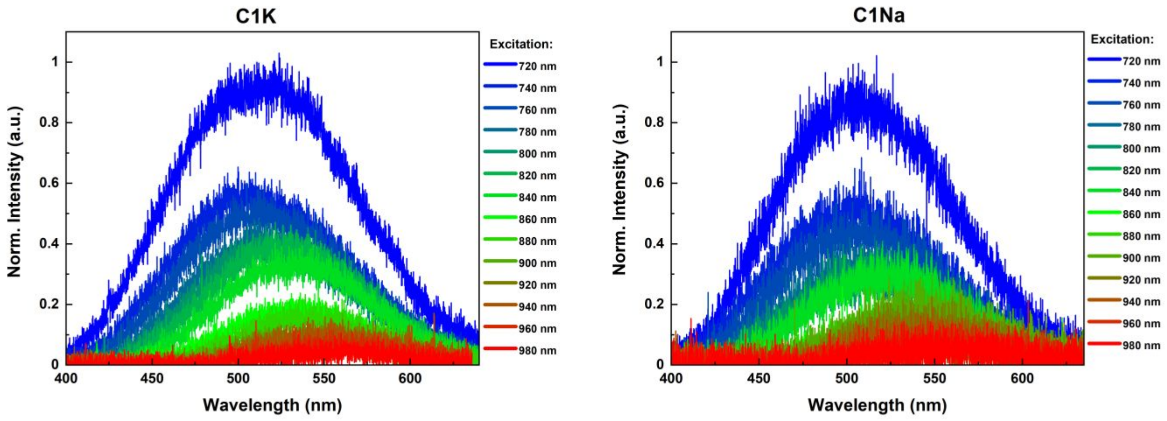

Figure S4. TPE FL spectra of pure PDs upon excitation in the wide wavelength range.

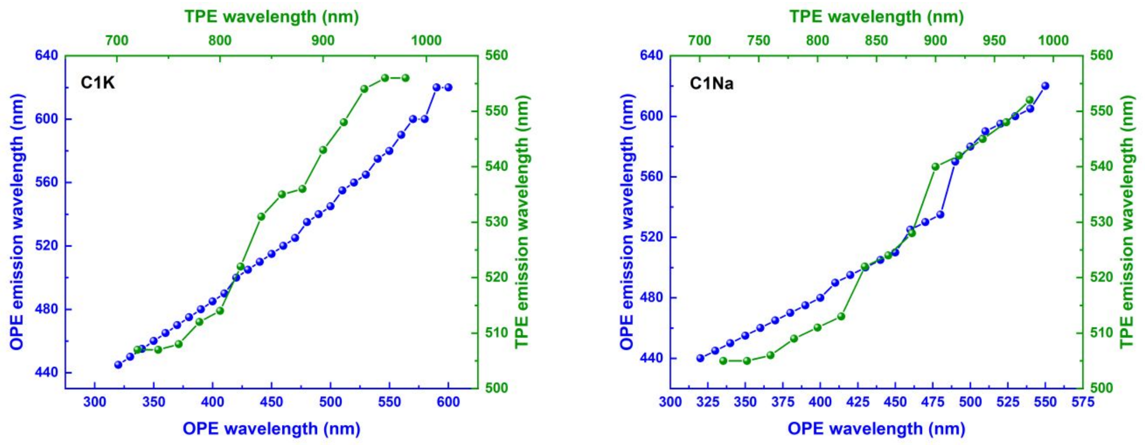

Figure S5. Excitation-dependent TPE fluorescence of pure PDs. Correlations between OPE and TPE wavelengths and emission maxima.

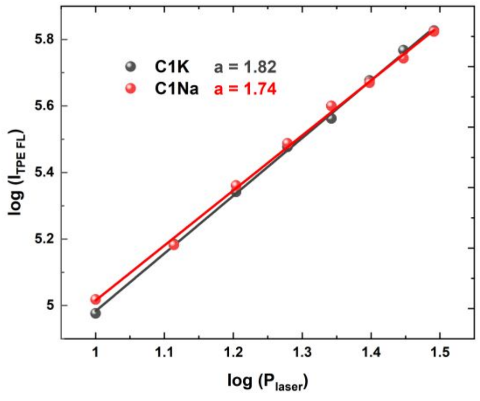

Figure S6. Log ($I_{TPE\ FL}$) *vs.* log (P_{laser}) for pure PDs with the slope values.

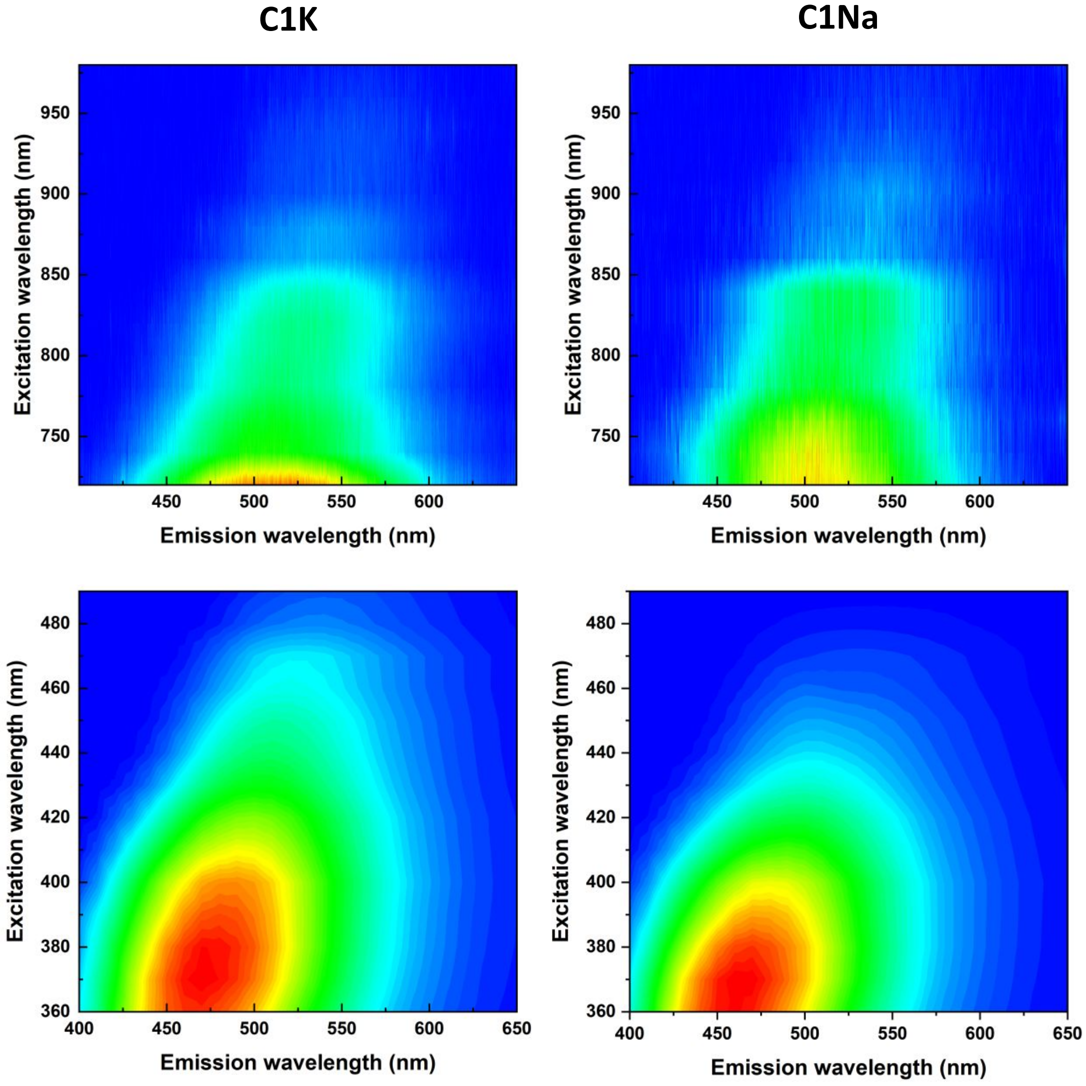

Figure S7. OPE (lower) and TPE (upper) excitation-emission maps of free PDs.

Two-photon absorption cross-section of reference dye

Table S2. Two-photon absorption cross-section values of fluorescein in alkalic solution as reference sample – results were reported by Makarov *et al.*[2]

Excitation wavelength (nm)	Two-photon absorption cross-section (GM)
**720**	21
**740**	30
**760**	45
**780**	45
**800**	36
**820**	28
**840**	13
**860**	12
**880**	12
**900**	12
**920**	14
**940**	15
**960**	16
**980**	16

Photobleaching resistance

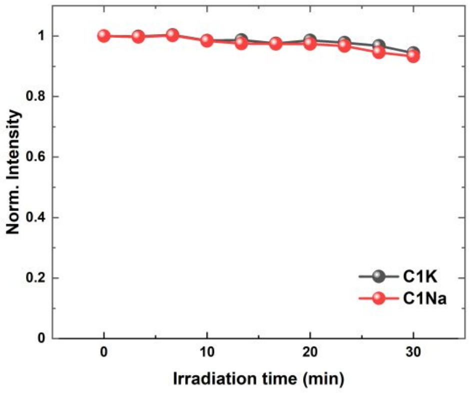

Figure S8. Changes in the fluorescence intensity as a function of laser irradiation time.

Enhanced one-photon excited fluorescence of PDs

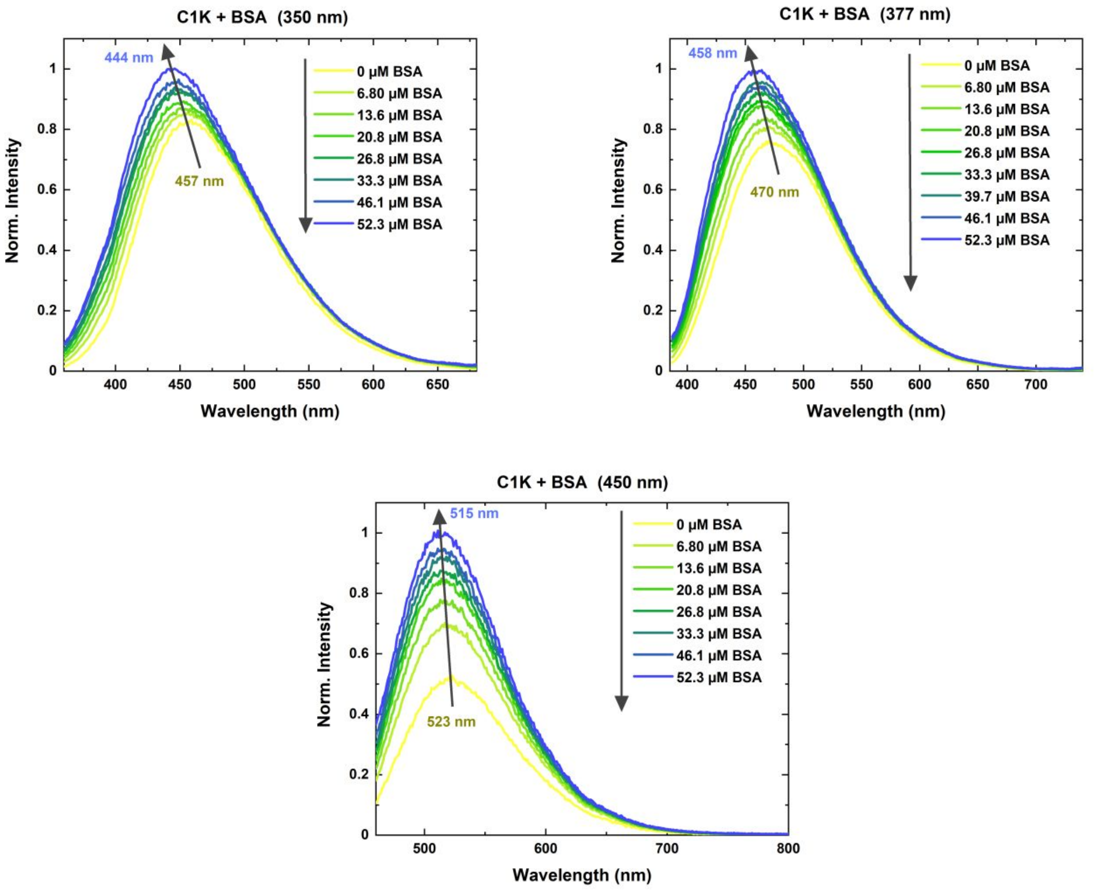

Figure S9. Evolution of BSA-induced enhanced OPE fluorescence of C1K-including systems in aqueous media (Mili-Q) as representative examples. Three excitation wavelengths are also indicated.

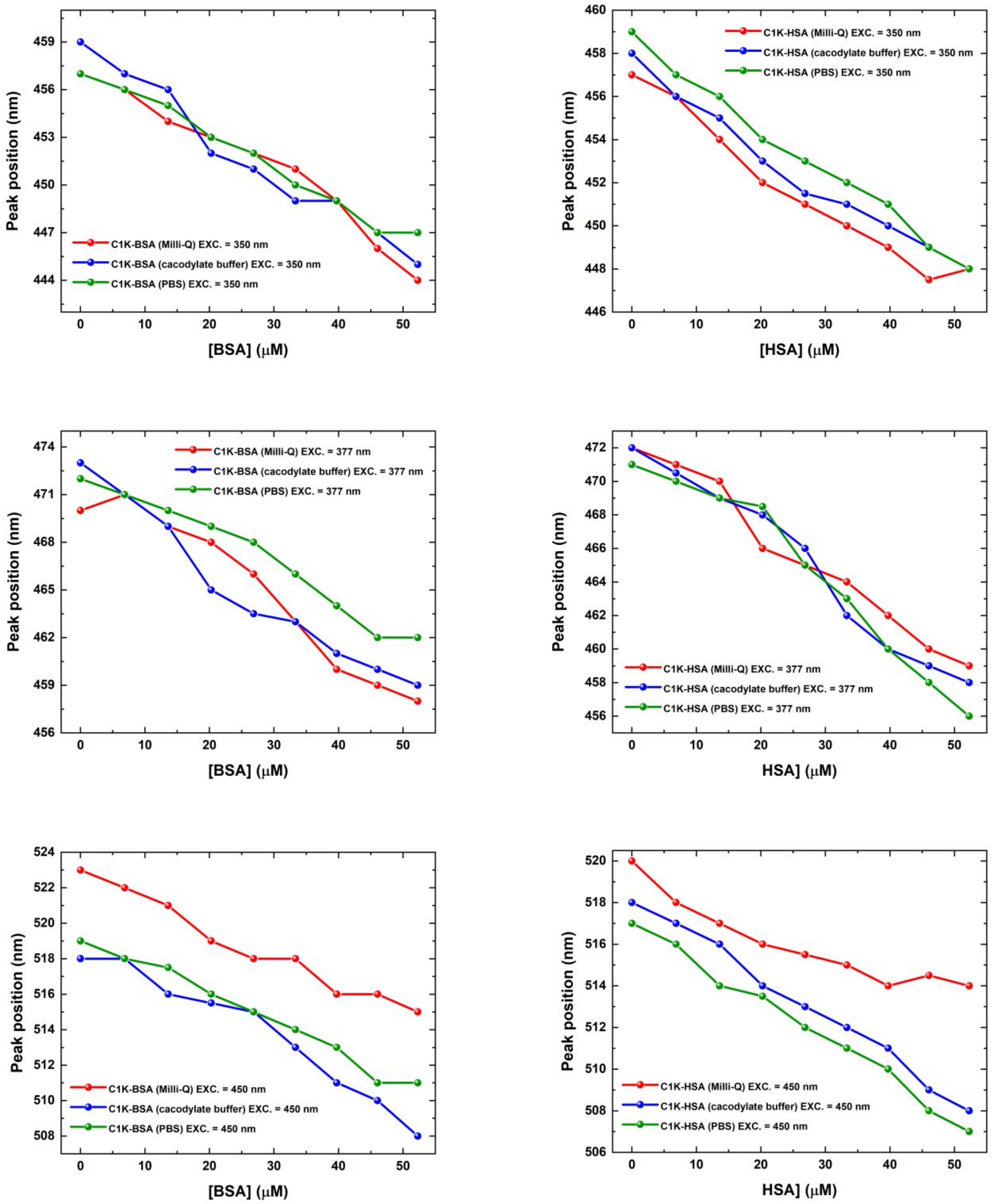

Figure S10. Evolution of peak positions in albumin-induced enhanced OPE fluorescence of C1K-including systems in different aqueous media: Milli-Q (red), cacodylate buffer (blue), and PBS (green). Each excitation wavelength is plotted separately.

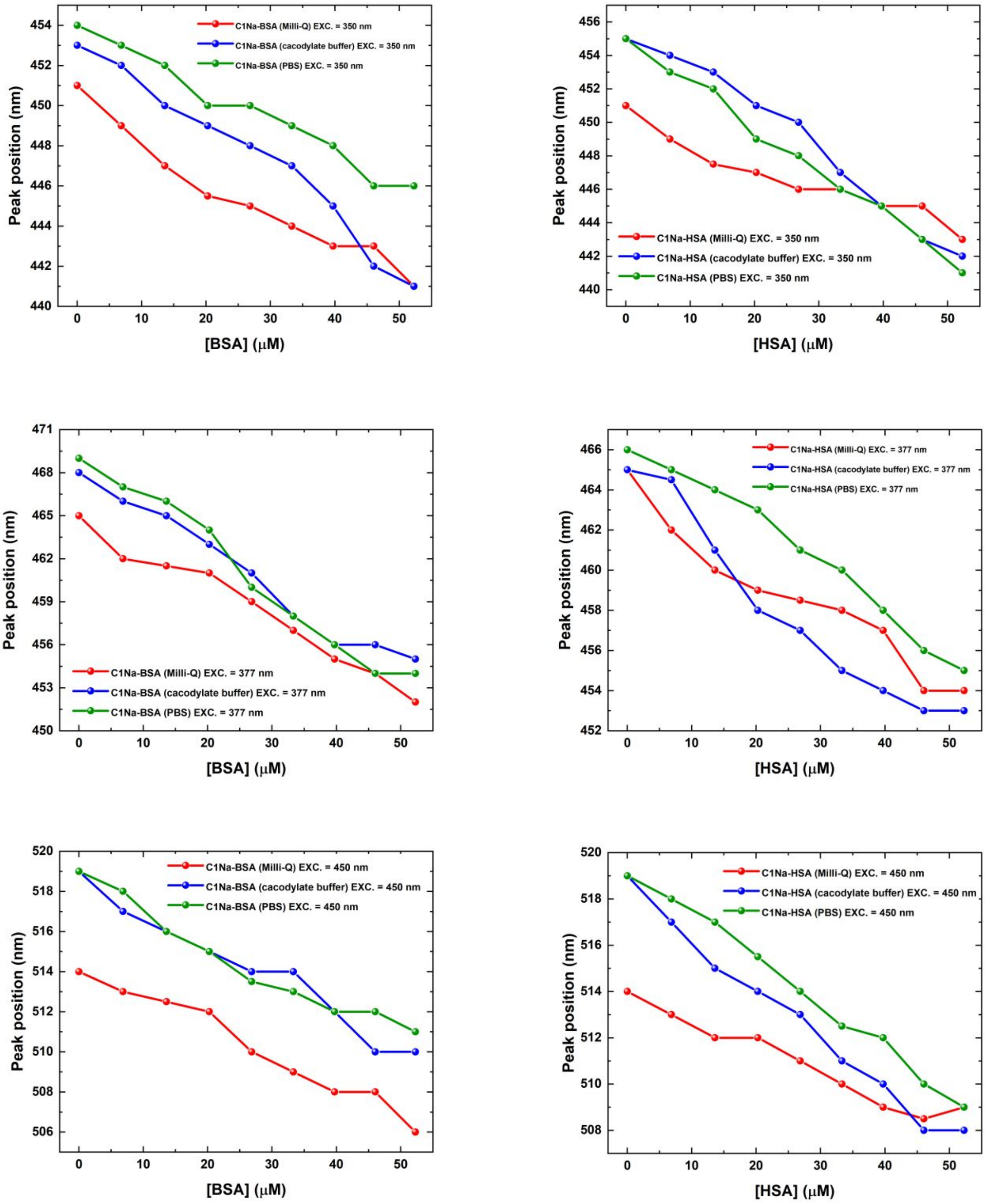

Figure S11. Evolution of peak positions in albumin-induced enhanced OPE fluorescence of C1Na-including systems in different aqueous media: Milli-Q (red), cacodylate buffer (blue), and PBS (green). Each excitation wavelength is plotted separately.

Linear optical properties of free albumins

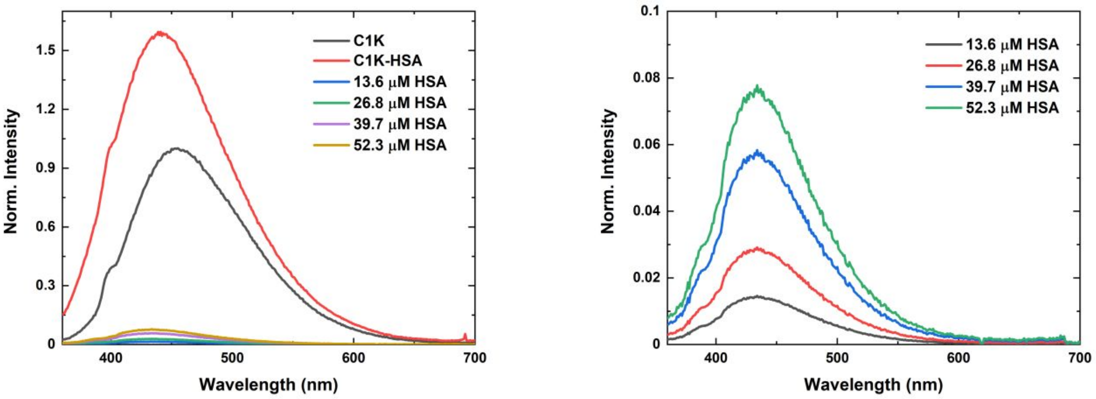

Figure S12. The comparison of OPE emission spectra for C1K, C1K-HSA, and free albumins at varying concentrations. The fluorescence intensity values were scaled with respect to the fluorescence signal of free PDs.

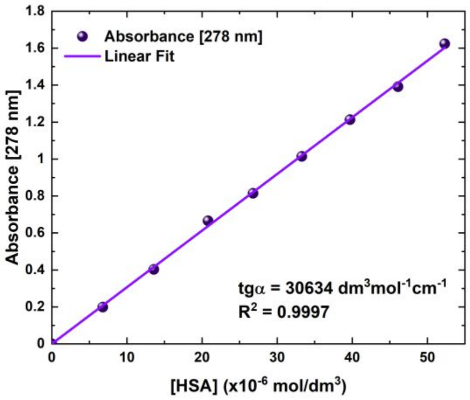

Figure S13. The linear correlation of absorbance values *vs.* concentrations of HSA.

Table S3. The normalized intensity values of C1K-HSA and free HSA.

	Normalized Intensity		Fluorescence maxima (nm)
	**C1K+52.3 µM HSA**	**52.3 µM HSA**	
**350 nm**	1.65	0.0766	434
**377 nm**	1.77	0.0545	449
**450 nm**	3.01	0.0350	505

Enhanced two-photon excited fluorescence of PDs

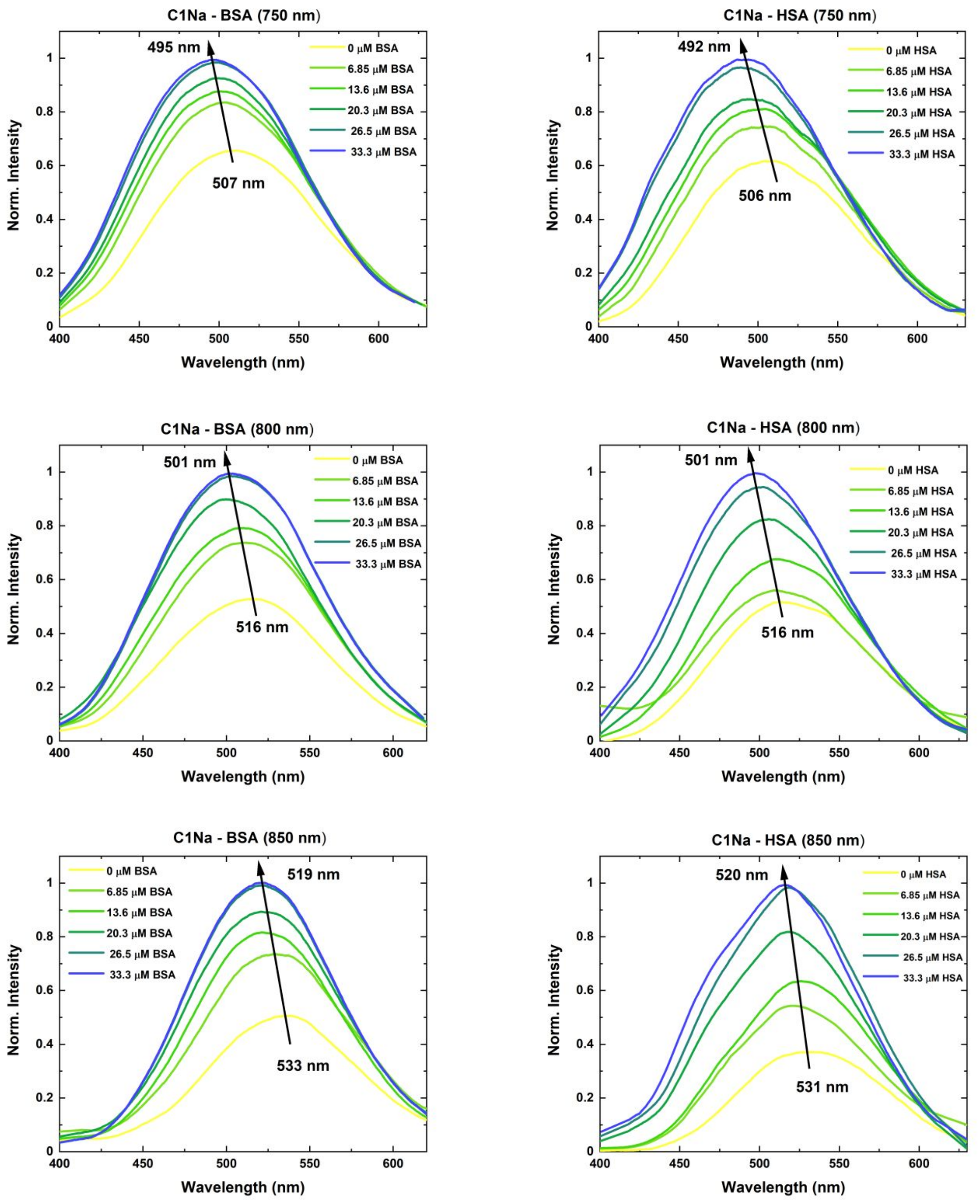

Figure S14. Evolution of protein-induced enhanced TPE fluorescence of C1Na-including systems. Three excitation wavelengths are also indicated.

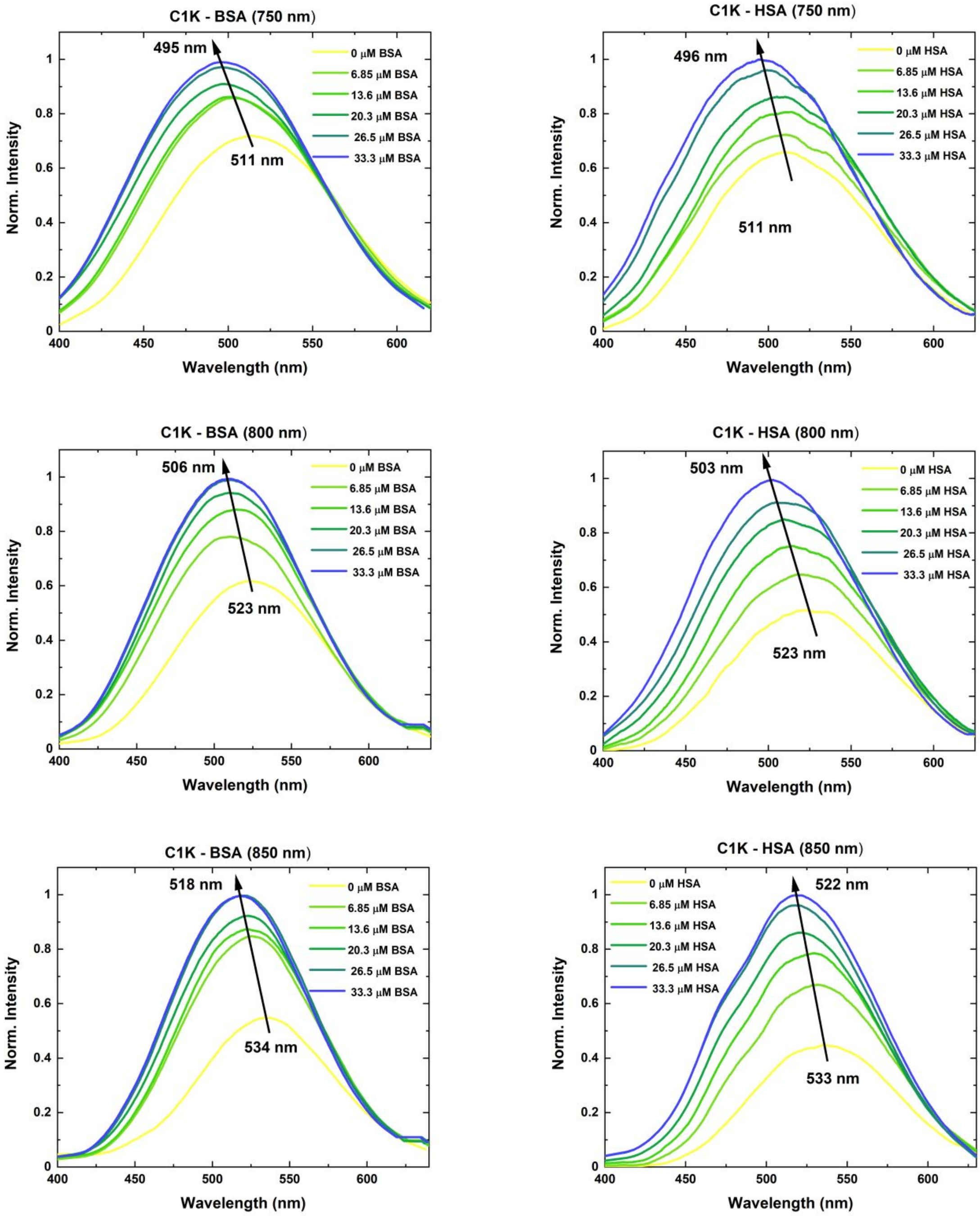

Figure S15. Evolution of protein-induced enhanced TPE fluorescence of C1K-including systems. Three excitation wavelengths are also indicated.

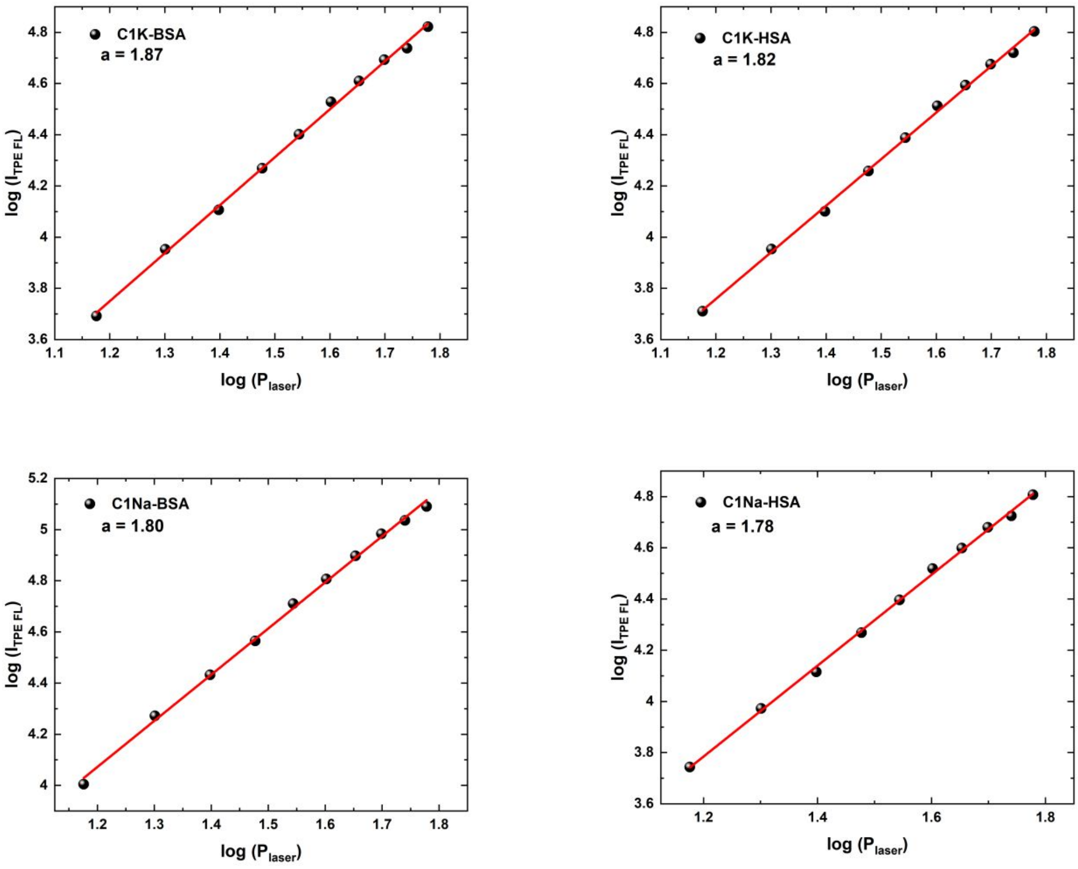

Figure S16. Log I *vs.* log P for protein-nanostructure assemblies.

The fluorescence enhancement coefficients

The fluorescence enhancement coefficients were estimated from OPE and TPE emission spectra, according to the following equation:

$$FEC(\lambda_{exc.}) = 100\% \frac{I_{complex} - I_{free\ PDs}}{I_{free\ PDs}}$$

where $FEC(\lambda_{exc.})$ denotes the fluorescence enhancement coefficient (%), $I_{complex}$ and $I_{free\ PDs}$ represent the integrated fluorescence intensity for a protein-nanostructure complex and unbound PDs (a.u.), respectively.

Table S4. The FEC parameters (%), calculated from OPE fluorescence spectra for samples in Mili-Q, cacodylate buffer, and PBS.

	FEC (%)			
	**C1K-BSA**	**C1K-HSA**	**C1Na-BSA**	**C1Na-HSA**
**350 nm**	29.8±1.3<br>(36.2±1.4)<br>(35.4±1.4)	66.9±2.7<br>(58.5±2.3)<br>(60.6±2.4)	63.4±2.5<br>(51.5±2.1)<br>(57.5±2.3)	119.2±6.1<br>(93.1±4.7)<br>(100.5±5.1)
**377 nm**	34.4±1.4<br>(36.6±1.5)<br>(36.7±1.5)	73.8±3.0<br>(61.1±2.4)<br>(65.6±2.6)	64.0±2.6<br>(56.2±2.2)<br>(60.2±2.4)	131.5±6.0<br>(112.7±5.2)<br>(111.6±5.1)
**450 nm**	91.2±3.2<br>(83.2±2.9)<br>(85.7±3.0)	110.2±3.9<br>(110.6±3.9)<br>(109.9±3.9)	152.6±5.0<br>(147.3±4.9)<br>(144.7±4.8)	276.3±10.8<br>(179.5±7.0)<br>(201.6±7.9)

Table S5. The FEC parameters (%), calculated from TPE fluorescence spectra.

	FEC (%)			
	**C1K-BSA**	**C1K-HSA**	**C1Na-HSA**	**C1Na-BSA**
**750 nm**	44.6±2.2	60.6±3.0	68.0±3.4	55.5±3.0
**800 nm**	66.6±3.3	100.5±5.0	96.4±4.8	88.4±4.6
**850 nm**	84.2±4.2	136.8±6.8	175.3±8.8	93.8±4.9

Absolute fluorescence quantum yields

To quantify how albumins improve fluorescence properties of PDs, the absolute fluorescence quantum yields (FQYs) were estimated, as shown below:[3-4]

$$FQY = \frac{S_2 - S_3}{S_0 - S_1}$$

where *FQY* is the calculated fluorescence quantum yield (%), S_2 denotes the integrated fluorescence intensity of sample (a.u.), S_0 and S_1 are the integrated intensity of an excitation peak in the absence and presence of a sample (a.u.), respectively, S_3 denotes the background in the emission region (a.u.);

Table S6. The FQYs of **free** and **protein-treated** PDs.

	C1K	C1K-BSA	C1K-HSA	C1Na	C1Na-HSA	C1Na-BSA
**FQYs (%)**	7.20	9.58	12.4	5.70	10.5	8.55

Two-photon absorption cross-section and brightness

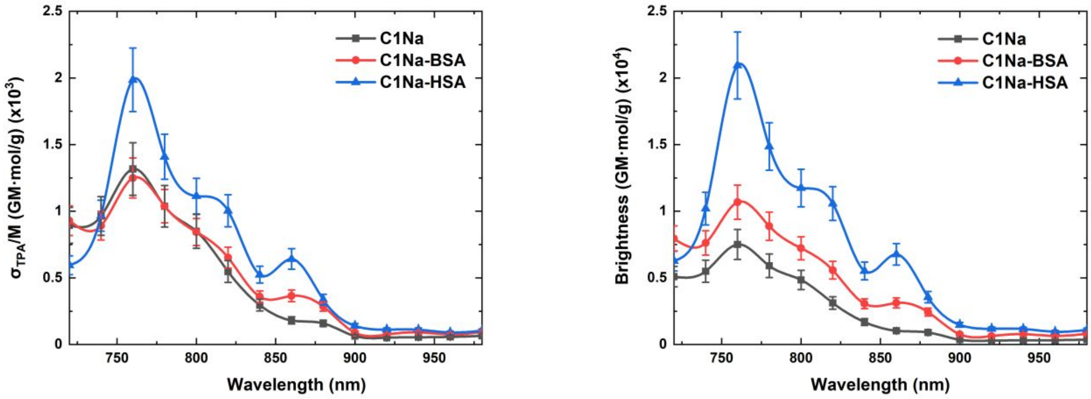

Figure S17. Molar-weight scaled TPA cross-section spectra of free and protein-including C1Na systems and their corresponding two-photon brightness spectra.

Fluorescence decays

All fluorescence decays were fitted with commercially available analysis software (SPCImage, Becker&Hickl), using the tri-exponential equation as follows:

$$I(t) = I_0 + A_1 exp\left(\frac{t - t_0}{\tau_1}\right) + A_2 exp\left(\frac{t - t_0}{\tau_2}\right) + A_3 exp\left(\frac{t - t_0}{\tau_3}\right)$$

where *I(t)* is the fluorescence intensity (a.u.), *t* represents time (ns), τ_1, τ_2, τ_3 denote lifetime components, and A_1, A_2, and A_3 are the relative decay amplitudes.

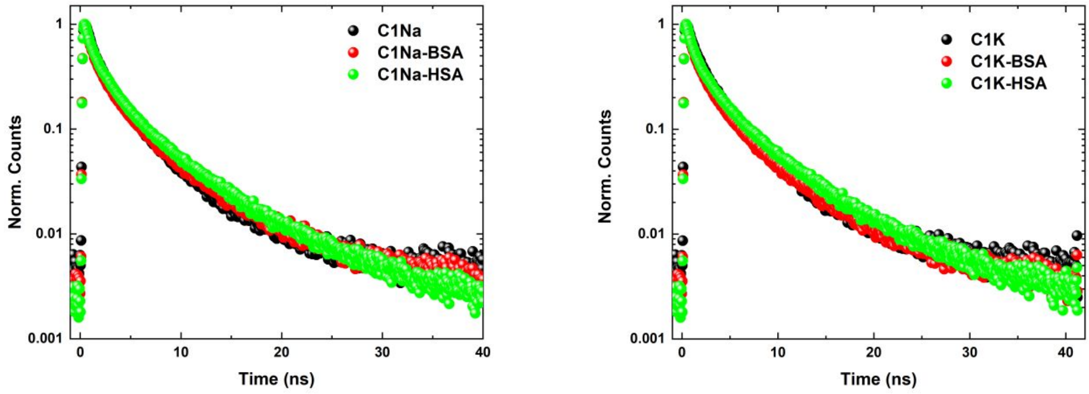

Figure S18. Normalized fluorescence decays of free and protein-including PDs.

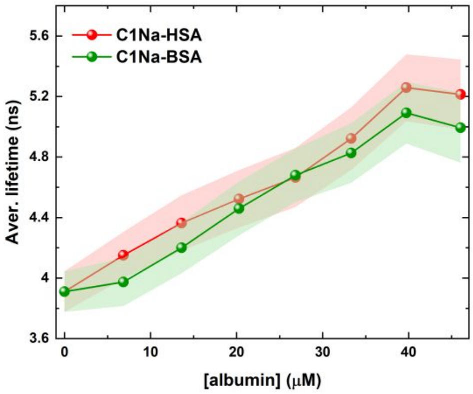

Figure S19. Evolution of fluorescence decays of PDs as a function of protein concentration. Red and green areas correspond to the error bars.

Stoichiometry

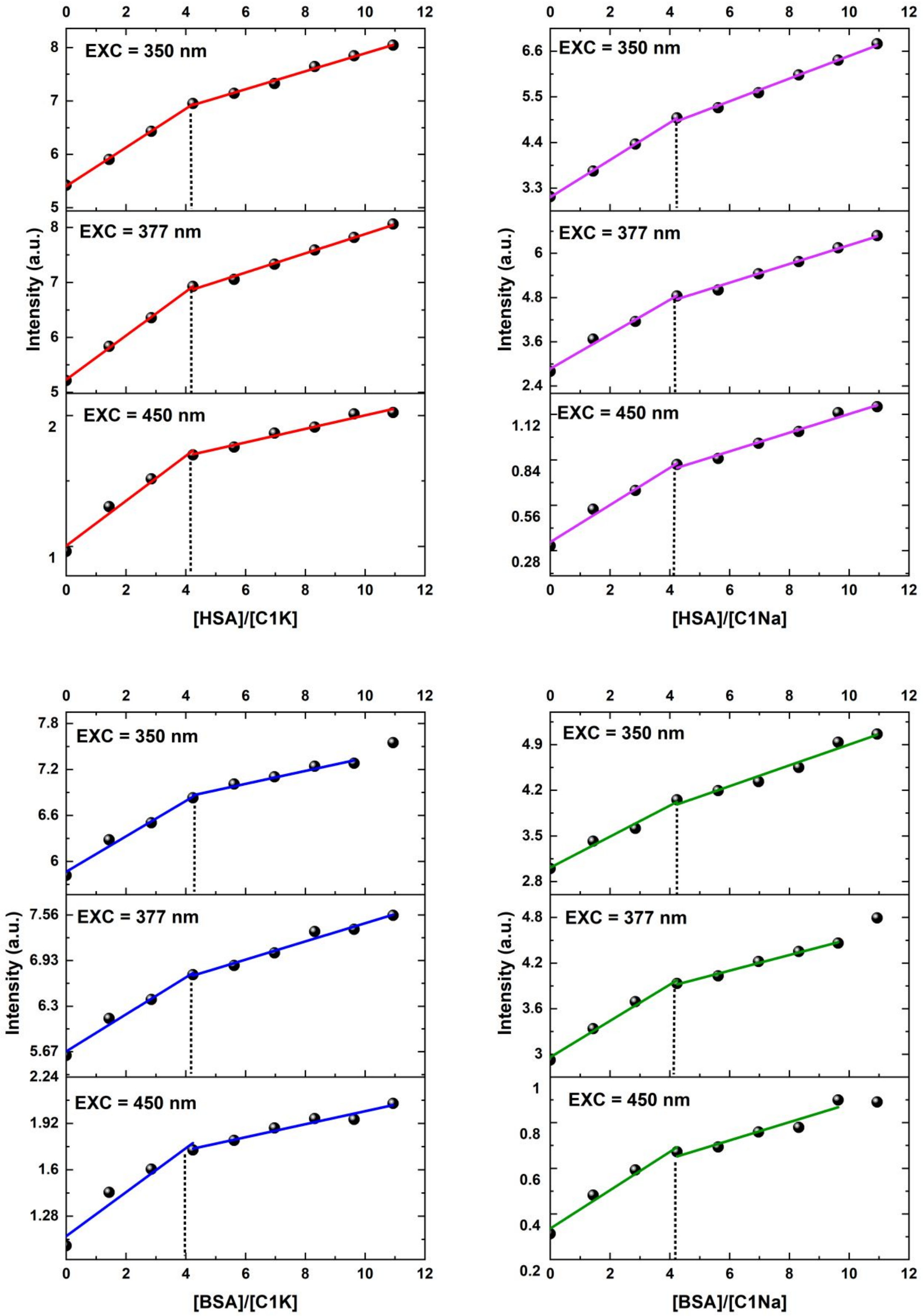

Figure S20. Stoichiometry determination derived from the OPE fluorescence titrations.

Circular dichroism

UV circular dichroism (CD) spectra of albumins and their corresponding bioconjugates at different luminophore/protein ratios were recorded, keeping constant concentration of albumins. To stimulate the inert environment, the sample chamber was deoxygenated with dry nitrogen, these conditions were held during the experiment. Spectroscopic measurements were performed, following the guidance provided by Norma J. Greenfield.[5] Additionally, pure PDs' dispersions were also measured to verify their chiroptical activity. Each CD spectrum were averaged after five accumulations. Afterward, as-obtained CD spectra were analysed with the K2D3 software to compute the secondary protein structure of native and bound albumins, as described by Caroline Louis-Jeune *et al.*[6].

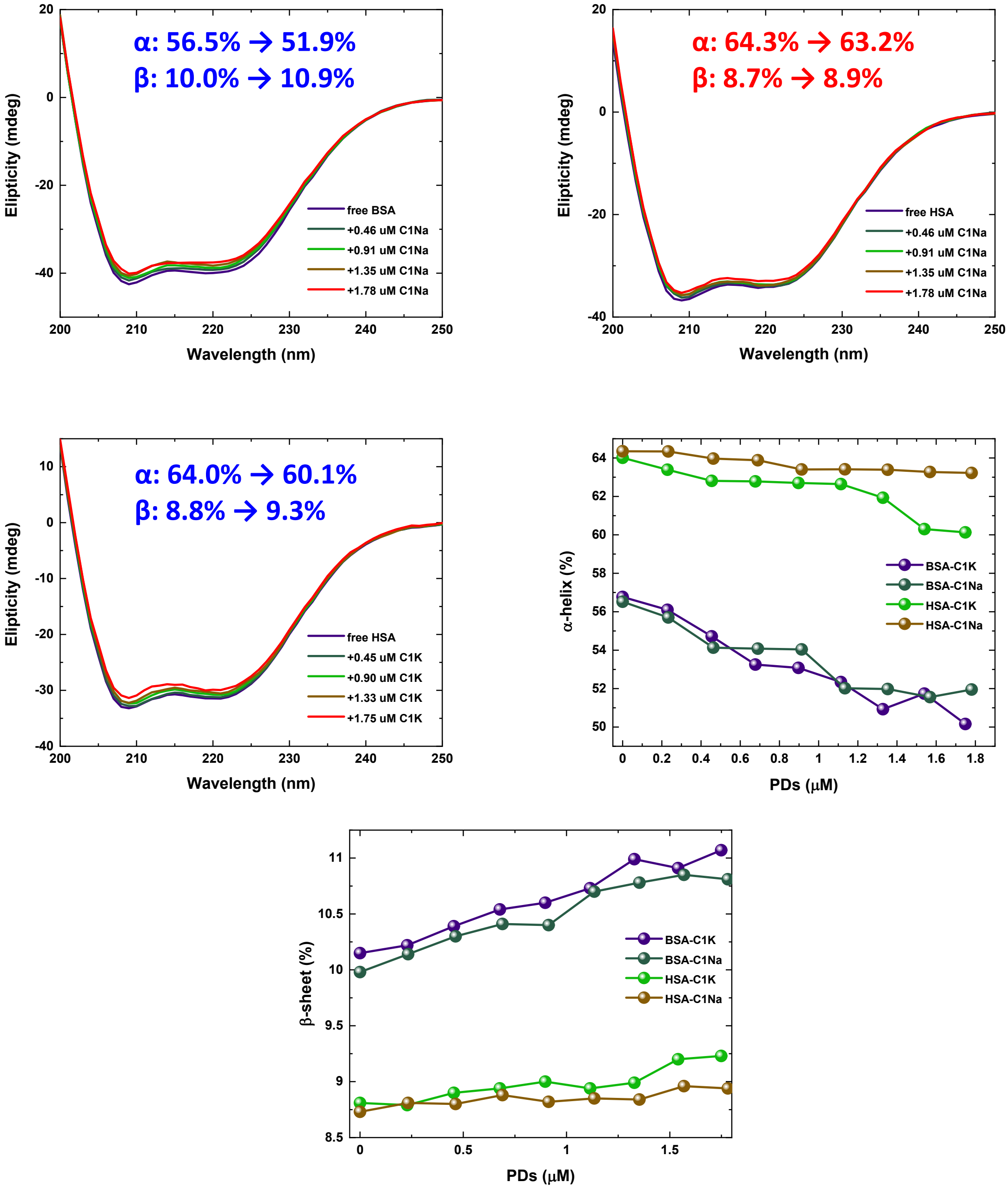

Figure S21. Changes in CD spectra of free proteins upon the titration with PDs dispersions. Evolution of the secondary protein structure as a function of PDs concentration.

Fourier-transform infrared spectra

The attenuated total-reflectance Fourier-transform infrared spectra (ATR-FTIR) of native proteins, pure PDs, and their conjugates were recorded in two different media, such as: Milli-Q and heavy water. All spectroscopic measurements and post-data treatment (*incl.* blank sample and nanostructure corrections) were performed, as reported in Huayan Yang *et al*.[7]

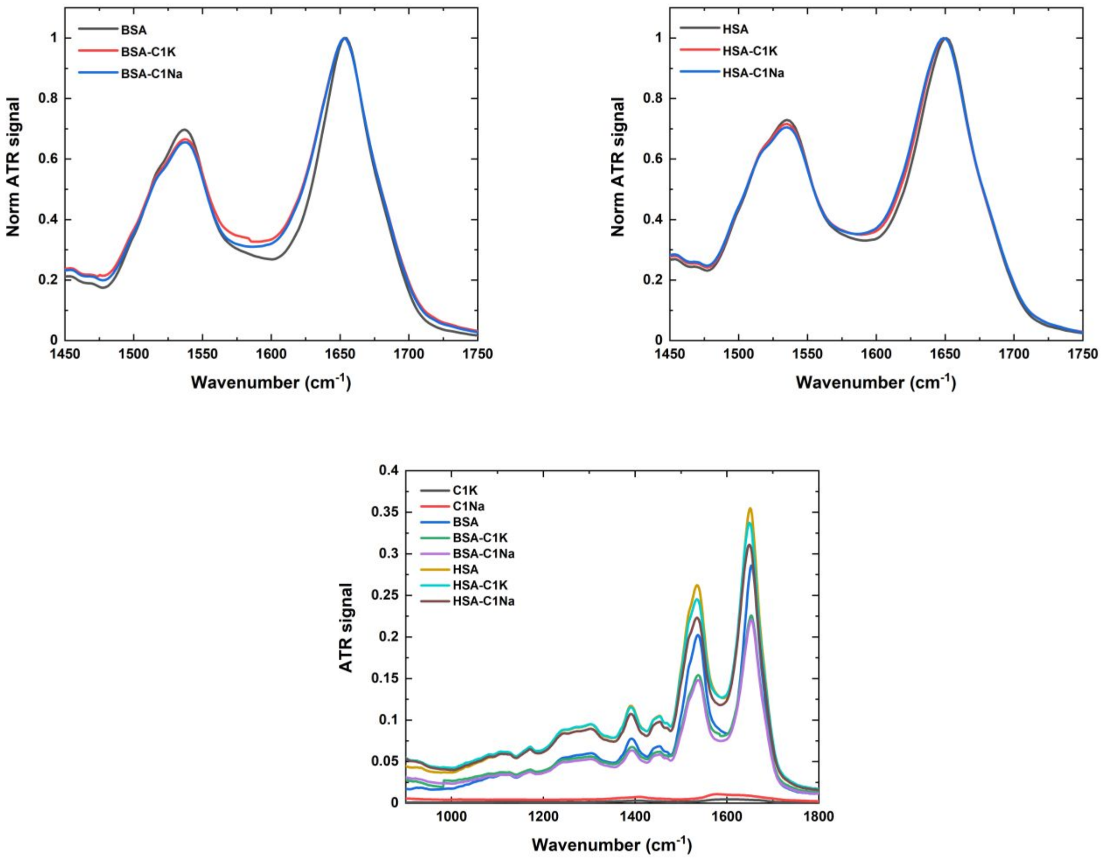

Figure S22. Normalized Amide I and II bands of native proteins and conjugates. The ATR-FTIR spectra in the wavenumber region.

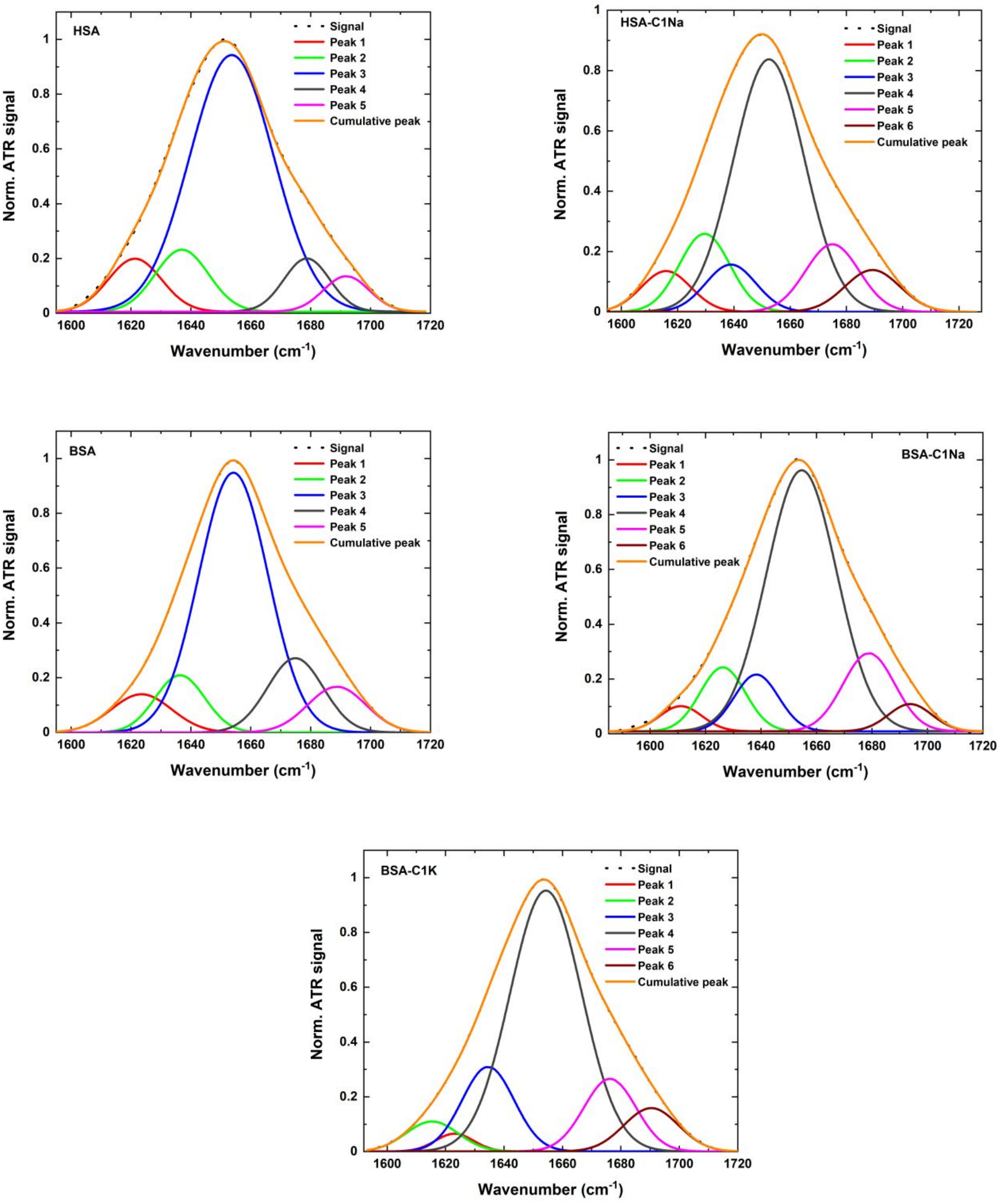

Figure S23. The Gaussian deconvolution of Amide I bands of native proteins and their PDs-including conjugates.

Table S7. Band assignments of peak components and their contribution in native proteins and conjugates.

Peaks	Sample	Peak position (cm^{-1})	Band assignments	Content (%)
1	**BSA**	1623.5	β-sheet	7.8%
2		1636	Random coil	9.5%
3		1654	**α-helix**	**60.1%**
4		1674	β-turn	13.8%
5		1688	β-antiparallel	8.9%
1	**BSA-C1Na**	1611	Intermolecular β-sheet	3.5%
2		1626	β-sheet	9.6%
3		1638	Random coil	8.4%
4		1654.5	**α-helix**	**61.1%**
5		1679	β-turn	13.4%
6		1694	β-antiparallel	3.9%
1	**BSA-C1K**	1615	Intermolecular β-sheet	2.2%
2		1623	β-sheet	5.0%
3		1634.5	Random coil	11.4%
4		1654	**α-helix**	**59.4%**
5		1676	β-turn	12.0%
6		1690	β-antiparallel	7.5%
1	**HSA**	1621	β-sheet	8.9%
2		1637	Random coil	11.4%
3		1653.5	**α-helix**	**64.9%**
4		1679	β-turn	9.7%
5		1691	β-antiparallel	5.1%
1	**HSA-C1K**	1615	Intermolecular β-sheet	5.1%
2		1628	β-sheet	12.6%
3		1639	Random coil	14.5%
4		1653	**α-helix**	**59.9%**
5		1674	β-turn	14.9%
6		1690	β-antiparallel	9.0%
1	**HSA-C1Na**	1615	Intermolecular β-sheet	4%
2		1629.5	β-sheet	12.5%
3		1639	Random coil	9.5%
4		1652.5	**α-helix**	**59%**
5		1675	β-turn	12%
6		1689	β-antiparallel	8%

SEC-GPC experiments

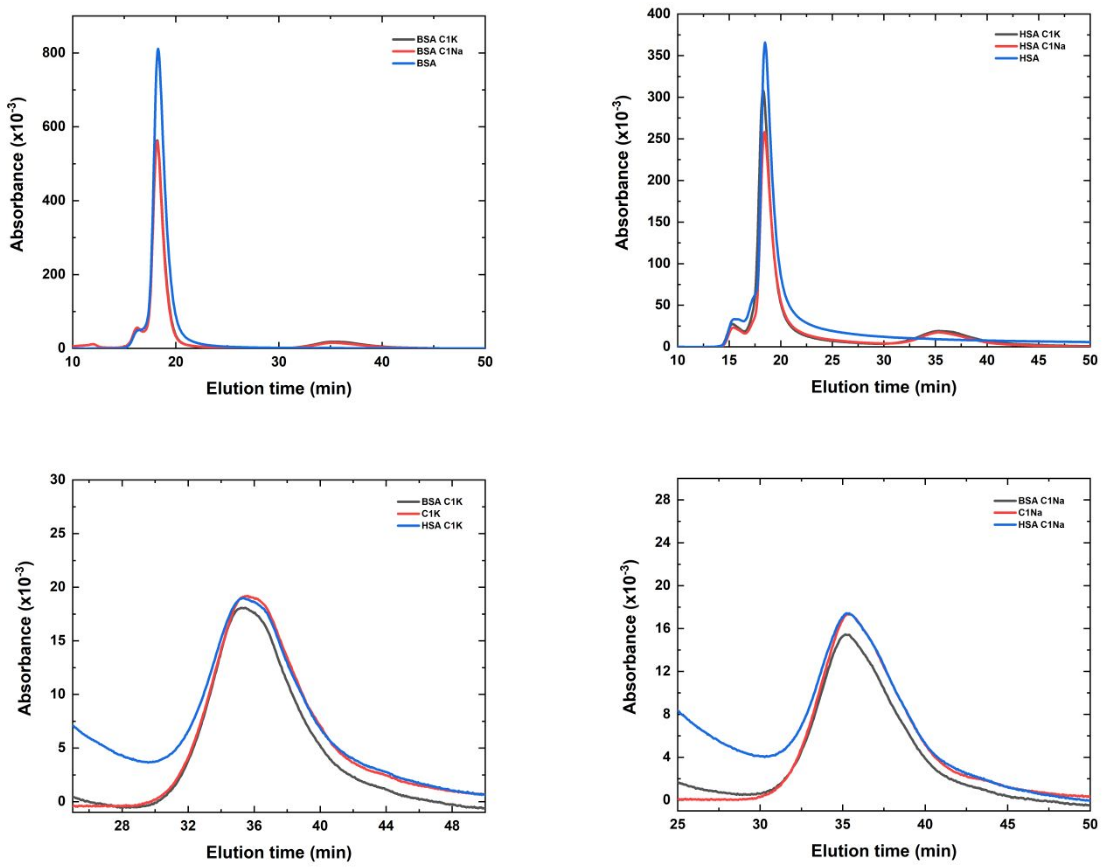

Figure S24. The GPC chromatograms of albumin and albumin-PDs samples as a function of elution time. Comparison of GPC signals for free PDs and albumin-including composites.

Table S8. The hydrodynamic diameter values of BSA for different compositions, where $D_{hydr.}$ is the hydrodynamic diameter (nm) and SD denotes the standard deviation (nm).

Sample	BSA	C1Na-BSA	C1K-BSA
**$D_{hydr.}$±SD (nm)**	8.3±2.4	8.6±2.7	8.5±2.7

Thermal stability of PDs

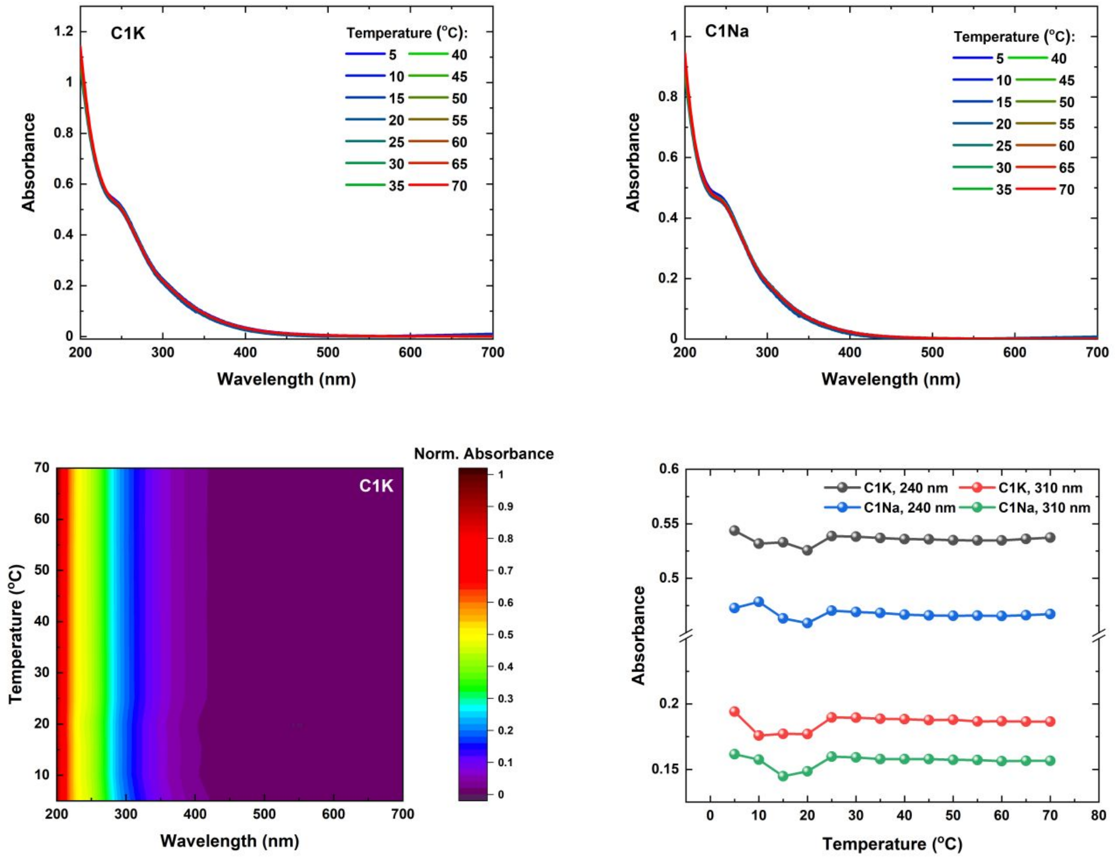

Figure S25. The UV-Vis extinction spectra of C1K and C1Na at varying temperature values (top). The normalized temperature-dependent extinction map of C1K (bottom left). The evolution of absorbance values at 240 and 310 nm as a function of temperature (bottom right).

Temperature-dependent one-photon excited emission

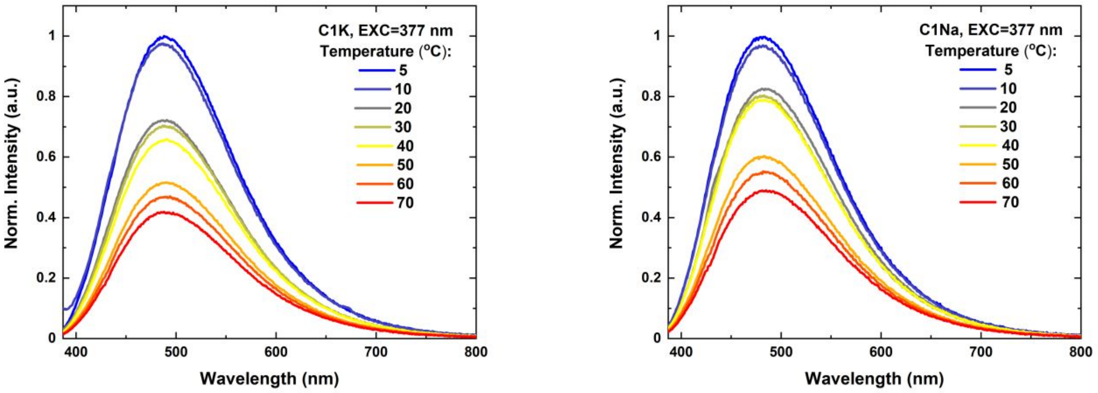

Figure S26. The OPE fluorescence spectra of both PDs ($\lambda_{exc.}$ = 377 nm) at varying temperature values as representative examples.

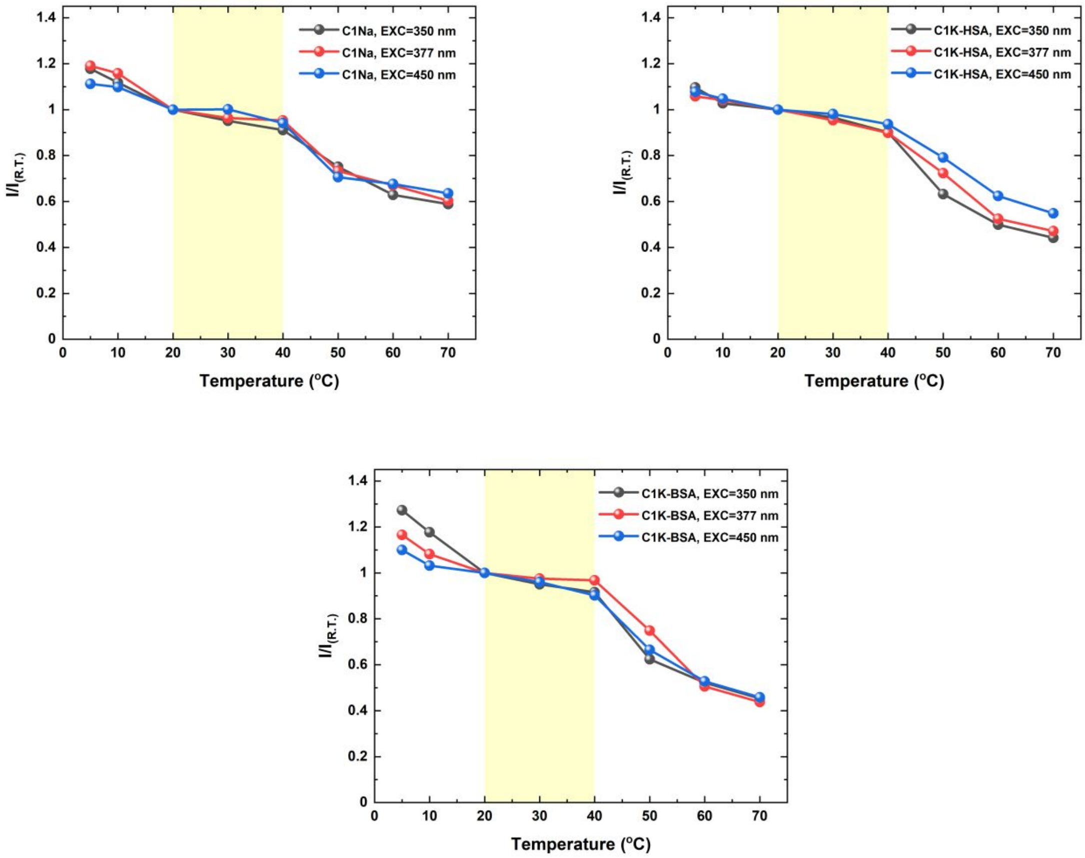

Figure S27. The evolution of the integrated OPE fluorescence intensity of C1Na and C1K-albumins samples as a function of temperature. As-calculated values were scaled by the integrated intensity arising from the emission spectrum at 20°C. The most crucial region for biological applications is highlighted as a yellow area.

pH effect on the fluorescence activity

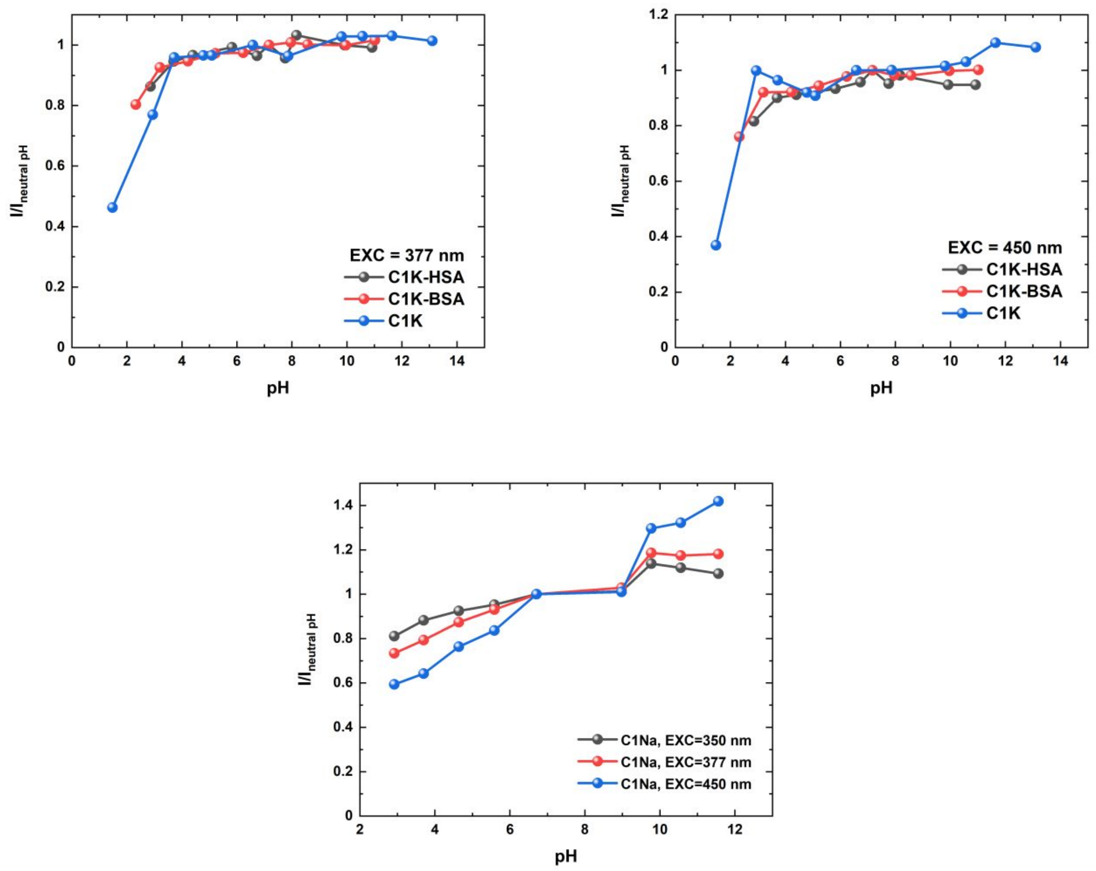

Figure S28. Changes in the integrated OPE fluorescence intensity *vs.* pH value for C1K-including examples (top) and C1Na (bottom).

Molecular interferents *vs.* OPE fluorescence

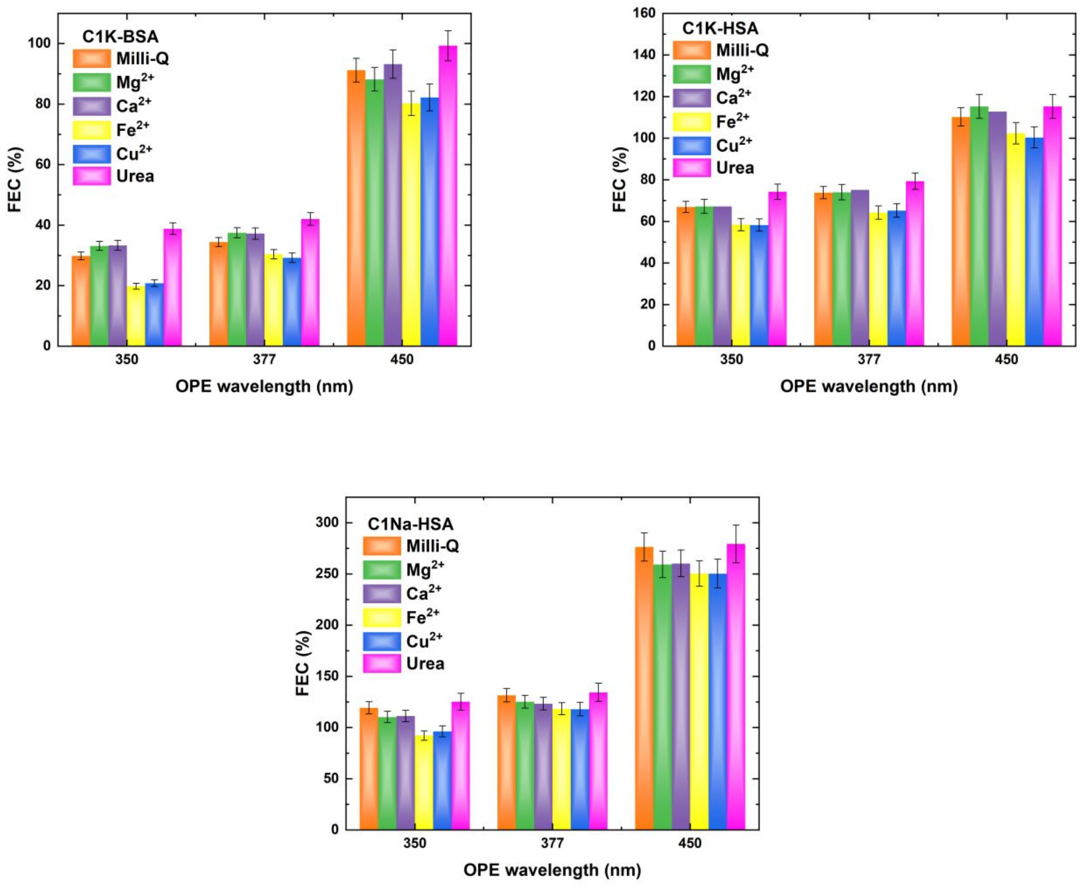

Figure S29. Variations in the FEC values showing the interferents' effect.

Selectivity towards proteins

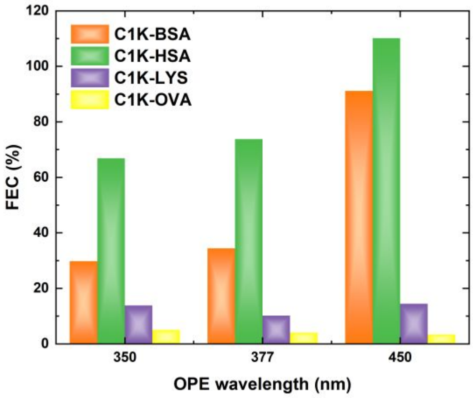

Figure S30. Selectivity of C1K towards various proteins based on the changing FEC values.

NMR characterization of PDs

Raw NMR data were interpreted based on the peaks' positions in the NMR spectroscopy handbooks,[8-9] identifying the most crucial sub-units.

^{13}C NMR (151 MHz, D_2O) δ: 215.4, 181.5, 180.7, 171.0, 69.9, 62.5, 57.4, 49.4, 30.2, 28.0, 27.7, 26.7, 23.2, 16.8

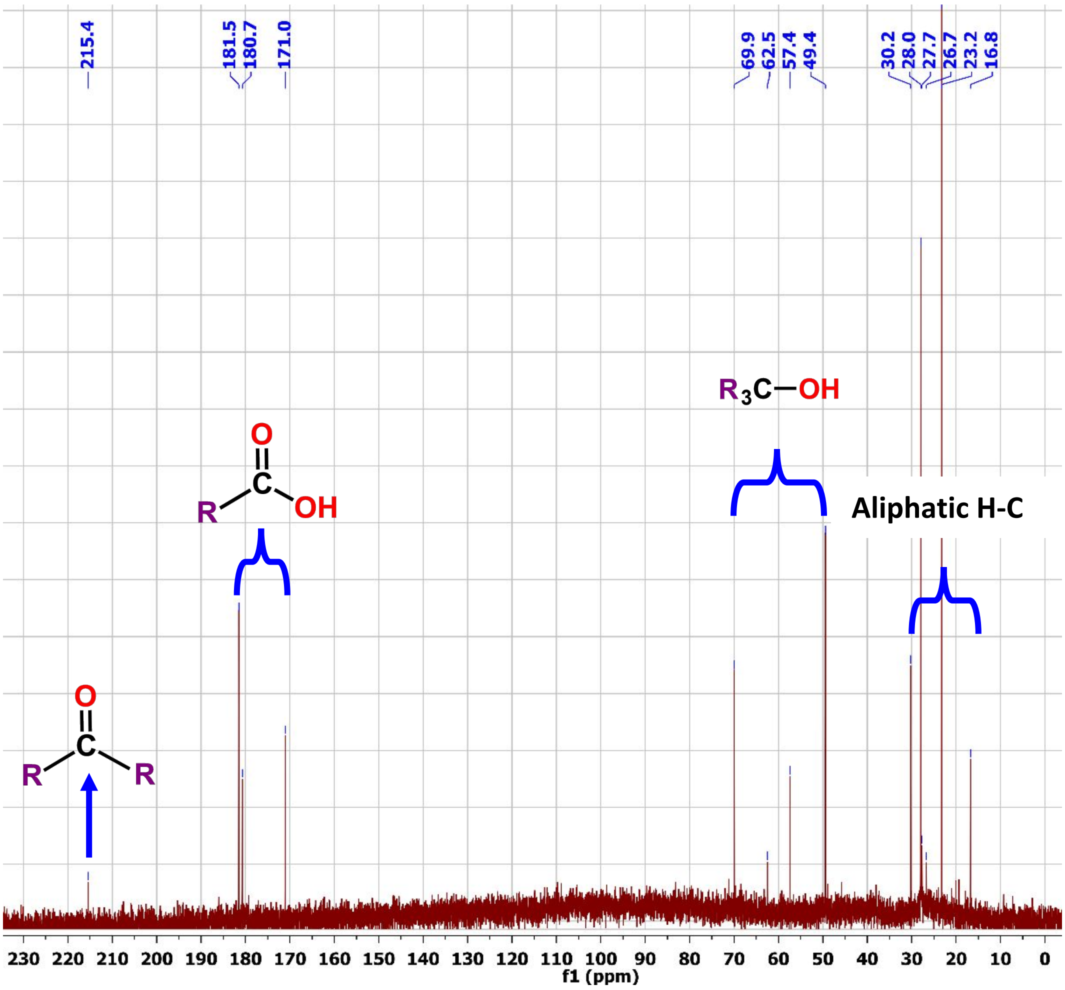

Figure S31. The ^{13}C NMR spectrum of C1Na. The major sub-groups of PDs are indicated with their peak positions above.

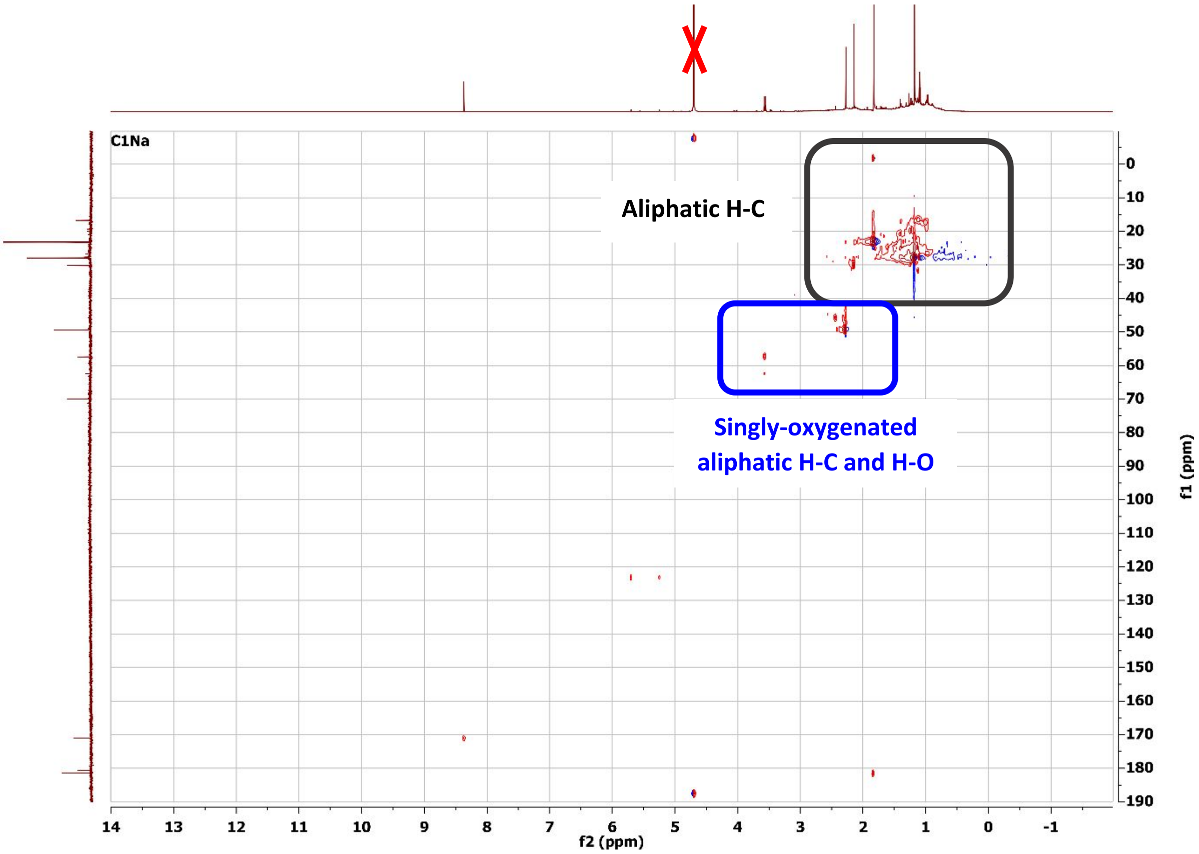

Figure S32. The HSQC NMR spectrum of C1Na. Two areas with the most common sub-groups are indicated with blue and black frames. The heavy water signal is marked with a red cross.

^{13}C NMR (151 MHz, D_2O) δ: 181.5, 180.7, 171.0, 69.9, 68.5, 51.2, 49.4, 28.0, 23.2, 20.0, 16.8.

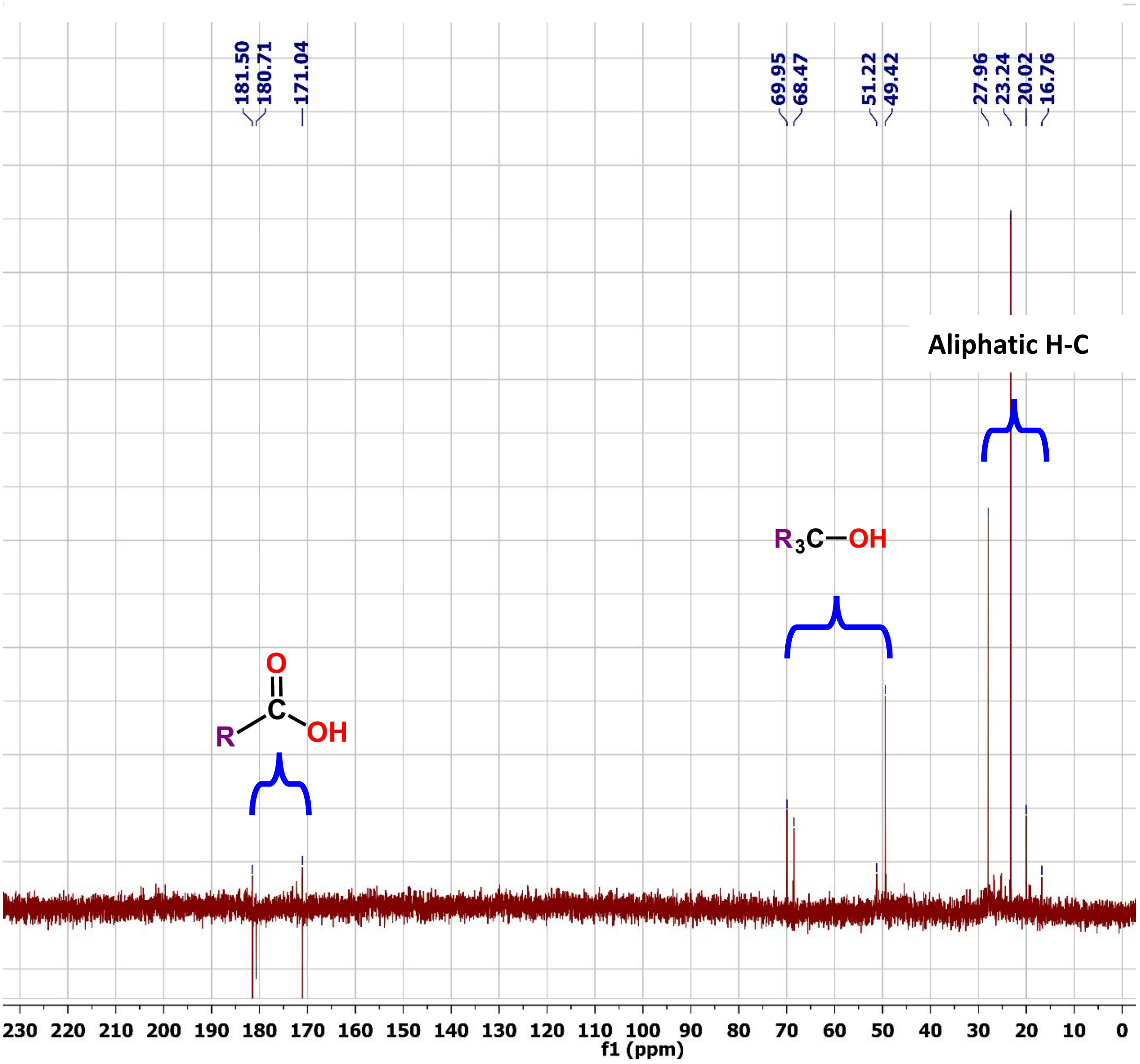

Figure S33. The ^{13}C NMR spectrum of C1K. The three major sub-groups of PDs are indicated with their peak positions above.

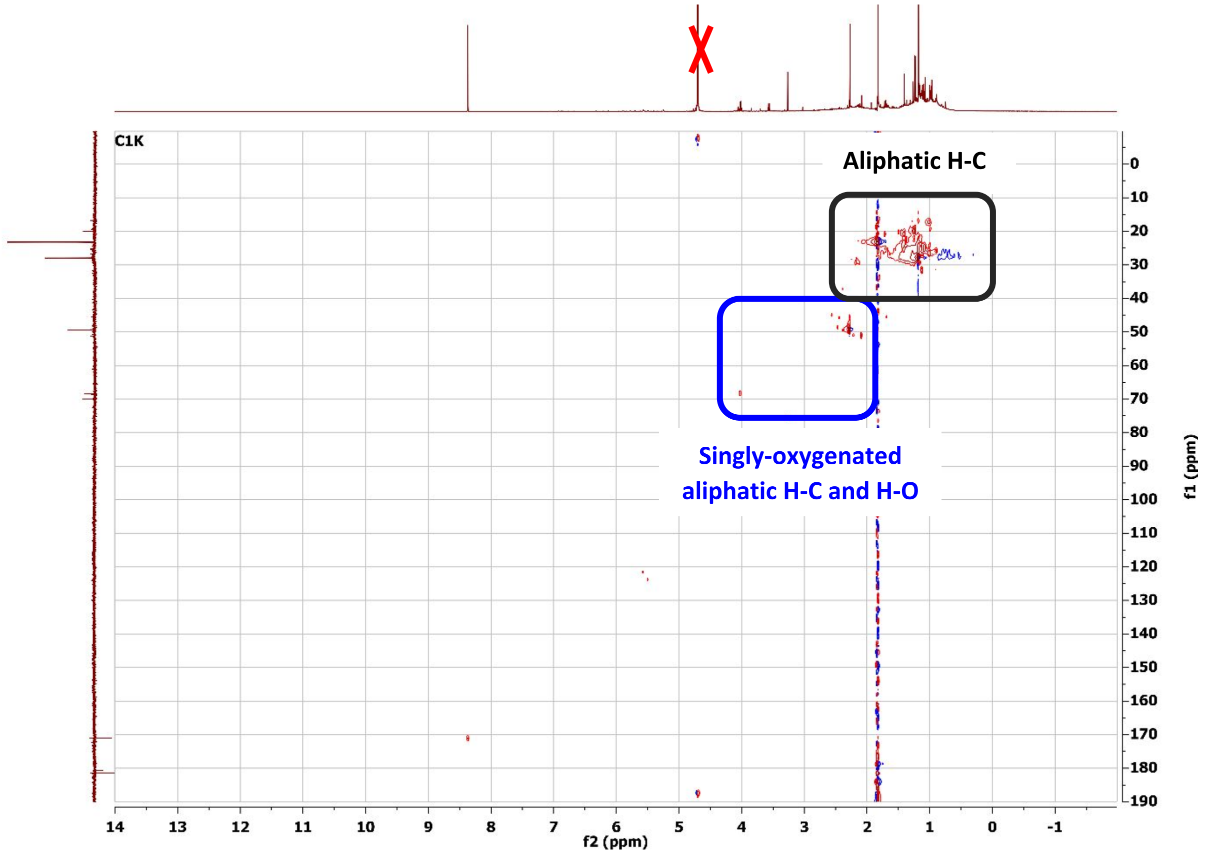

Figure S34. The HSQC NMR spectrum of C1K. Two areas with the most common sub-groups are indicated with blue and black frames. The heavy water signal is marked with a red cross.

Albumins' sensing

Table S8. LOD values of BSA and HSA.

	LOD (μM)			
	**C1K-BSA**	**C1K-HSA**	**C1Na-HSA**	**C1Na-BSA**
**350 nm**	4.3	4.7	3.2	4.6
**377 nm**	5.8	5.5	4.6	5.2
**450 nm**	6.8	6.8	5.4	4.4

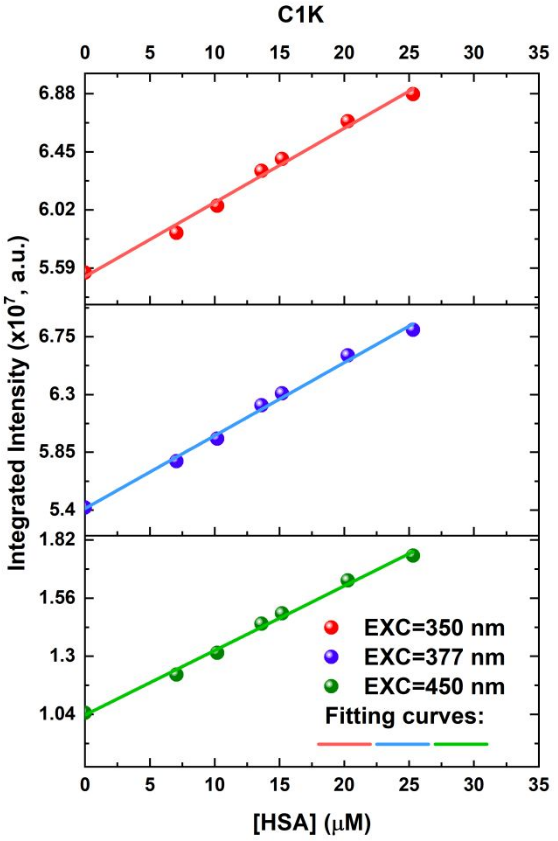

Figure S35. Representative calibration curves for determination of LODs of HSA using C1K as a probe.

Table S9. Regression curve characteristics, where **a** denotes the slope of the linear relation between the integrated fluorescence intensity of PDs and the concentration of albumins, σ is the standard error of the regression line. Both parameters were rounded.

	Calibration curve parameters			
	**C1K-BSA**<br>**a (x10^5)**<br>**σ (x10^2)**	**C1K-HSA**<br>**a (x10^5)**<br>**σ (x10^2)**	**C1Na-HSA**<br>**a (x10^5)**<br>**σ (x10^2)**	**C1Na-BSA**<br>**a (x10^5)**<br>**σ (x10^2)**
**350 nm**	4.3970<br>6.3	5.4857<br>8.6	6.7957<br>7.3	3.8207<br>5.8
**377 nm**	4.0056<br>7.8	5.6856<br>10	6.695<br>10	3.3964<br>6.3
**450 nm**	3.1793<br>7.2	2.8880<br>6.5	1.6583<br>3.0	1.7581<br>2.6

References:

1. Mucha, S. G.; Firlej, L.; Bantignies, J.-L.; Żak, A.; Samoć, M.; Matczyszyn, K., Acetone-derived luminescent polymer dots: a facile and low-cost synthesis leads to remarkable photophysical properties. *RSC Advances* **2020,** *10* (63), 38437-38445.
2. Makarov, N. S.; Drobizhev, M.; Rebane, A., Two-photon absorption standards in the 550–1600 nm excitation wavelength range. *Opt. Express* **2008,** *16* (6), 4029-4047.
3. Leyre, S.; Coutino-Gonzalez, E.; Joos, J. J.; Ryckaert, J.; Meuret, Y.; Poelman, D.; Smet, P. F.; Durinck, G.; Hofkens, J.; Deconinck, G.; Hanselaer, P., Absolute determination of photoluminescence quantum efficiency using an integrating sphere setup. *Review of Scientific Instruments* **2014,** *85* (12), 123115.
4. Boyer, J.-C.; van Veggel, F. C. J. M., Absolute quantum yield measurements of colloidal NaYF4: Er3+, Yb3+ upconverting nanoparticles. *Nanoscale* **2010,** *2* (8), 1417-1419.
5. Greenfield, N. J., Using circular dichroism spectra to estimate protein secondary structure. *Nature protocols* **2006,** *1* (6), 2876-2890.
6. Louis-Jeune, C.; Andrade-Navarro, M. A.; Perez-Iratxeta, C., Prediction of protein secondary structure from circular dichroism using theoretically derived spectra. *Proteins: Structure, Function, and Bioinformatics* **2012,** *80* (2), 374-381.
7. Yang, H.; Yang, S.; Kong, J.; Dong, A.; Yu, S., Obtaining information about protein secondary structures in aqueous solution using Fourier transform IR spectroscopy. *Nature protocols* **2015,** *10* (3), 382-96.
8. Jacobsen, N. E., *NMR data interpretation explained : understanding 1D and 2D NMR spectra of organic compounds and natural products*. 2017.
9. Silverstein, R. M.; Webster, F. X.; Kiemle, D., *Spectrometric Identification of Organic Compounds, 7th Edition*. Wiley: 2005.
